# NF-E2, FLI1 and RUNX1 collaborate at areas of dynamic chromatin to activate transcription in mature mouse megakaryocytes

**DOI:** 10.1038/srep30255

**Published:** 2016-07-26

**Authors:** Chongzhi Zang, Annouck Luyten, Justina Chen, X. Shirley Liu, Ramesh A. Shivdasani

**Affiliations:** 1 Department of Biostatistics and Computational Biology, Dana-Farber Cancer Institute and Harvard T.H. Chan School of Public Health, Boston, MA 02215, USA; 2 Center for Functional Cancer Epigenetics, Dana-Farber Cancer Institute, Boston, MA 02215, USA; 3 Department of Medical Oncology, Dana-Farber Cancer Institute, Boston, MA 02215, USA; 4 Department of Medicine, Harvard Medical School, Boston, MA 02115, USA; 5 Department of Pediatric Hematology/Oncology, Boston Children’s Hospital and Harvard Medical School, Boston, MA 02115, USA

## Abstract

Mutations in mouse and human *Nfe2*, *Fli1* and *Runx1* cause thrombocytopenia. We applied genome-wide chromatin dynamics and ChIP-seq to determine these transcription factors’ (TFs) activities in terminal megakaryocyte (MK) maturation. Enhancers with H3K4me2-marked nucleosome pairs were most enriched for NF-E2, FLI and RUNX sequence motifs, suggesting that this TF triad controls much of the late MK program. ChIP-seq revealed NF-E2 occupancy near previously implicated target genes, whose expression is compromised in *Nfe2*-null cells, and many other genes that become active late in MK differentiation. FLI and RUNX were also the motifs most enriched near NF-E2 binding sites and ChIP-seq implicated FLI1 and RUNX1 in activation of late MK, including NF-E2-dependent, genes. Histones showed limited activation in regions of single TF binding, while enhancers that bind NF-E2 and either RUNX1, FLI1 or both TFs gave the highest signals for TF occupancy and H3K4me2; these enhancers associated best with genes activated late in MK maturation. Thus, three essential TFs co-occupy late-acting *cis*-elements and show evidence for additive activity at genes responsible for platelet assembly and release. These findings provide a rich dataset of TF and chromatin dynamics in primary MK and explain why individual TF losses cause thrombopocytopenia.

Terminally differentiated megakaryocytes (MKs) assemble thousands of platelets *de novo* and release them into the circulation^1^,^2^. The high synthetic demand for thrombopoiesis requires coordinated expression of hundreds of MK-specific genes, reflecting cell- and stage-specific chromatin modulation and transcription factor (TF) binding. Defects in this response underlie some clinical states of platelet deficiency. The TFs FLI1 and RUNX1, for example, are causally implicated in disorders of thrombopoiesis: Paris-Trousseau syndrome (OMIM 188025) and familial platelet disorder with predisposition to acute myeloid leukemia (FDP-AML, OMIM 601399), respectively^3^,^4^,^5^.

These and other TFs are expressed in multiple blood lineages^6^,^7^, and cell-restricted transcriptional activity represents the outcome of their specific interactions with each other and with chromatin. Erythroid cells and MKs derive from a common bipotential progenitor^8^ and share expression of several TFs such as GATA1, NF-E2, TAL1, RUNX1, and FLI1. Mice lacking any of these TFs have various defects in erythropoiesis and thrombopoiesis, and may even die in utero^9^,^10^, with each TF controlling different genes at various stages in sibling cells. *Fli1* and *Nfe2* null mice lack circulating platelets, reflecting a late MK maturation arrest, reduced α-granules, and disorganized internal membranes in both cases; *Nfe2*^−/−^ MKs elaborate no proplatelets^3^,^11^,^12^,^13^,^14^,^15^. *Runx1* deficiency causes mild but significant MK deficits, including arrested maturation and thrombocytopenia^16^,^17^. These defects reflect the TFs’ earliest overall requirement and no transcriptional targets, alone or as a group, explain them fully. In the multipotent progenitor cell line HPC-7^18^ and in cultured human MKs^19^, some of the same TFs co-occupy regulatory regions more commonly than do solitary TFs or pairs. Five TFs in particular - GATA1, GATA2, RUNX1, FLI1 and TAL1- co-occupy many genomic sites in immature human MKs^19^ and analysis of cultured murine MKs reinforces the idea that these TFs prime MK genes in progenitor cells^20^. However, fewer than 1/3 of MK-specific genes showed nearby binding of this TF pentad, and the outcomes and chromatin states associated with TF binding in mature MKs remain unclear.

As the combinatorial basis for MK maturation and platelet release are not understood well, we reasoned that *cis*-regulatory regions activated late in MK maturation might hold useful clues. Dynamic changes in active chromatin can accurately identify enhancers that function at specific stages in cell differentiation^21^,^22^ and dimethylation of Lysine 4 on Histone 3 (H3K4me2), in particular, appears at both active promoters and enhancers^23^,^24^,^25^,^26^. Moreover, we previously showed^24^ that H3K4me2 profiles parallel those for H3K27ac, the histone modification most commonly considered to mark functional enhancers^21^,^22^,^23^. To investigate transcriptional control of platelet biogenesis, we examined genome-wide dynamics of H3K4me2-marked chromatin in young and terminally differentiated MK. In so doing, we identified significant activity of three TFs known to be necessary for platelet biogenesis *in vivo*: NF-E2, FLI1 and RUNX1. We then examined the separate and joint activities of these TFs in regulating a large proportion of genes expressed toward the end of the MK life cycle, coincident with platelet release. The sum of our original, high-quality data reveals individual TFs’ activities in thrombopoiesis.

## Results

### Identification of dynamic enhancers in differentiating MK

**Figure 1:**
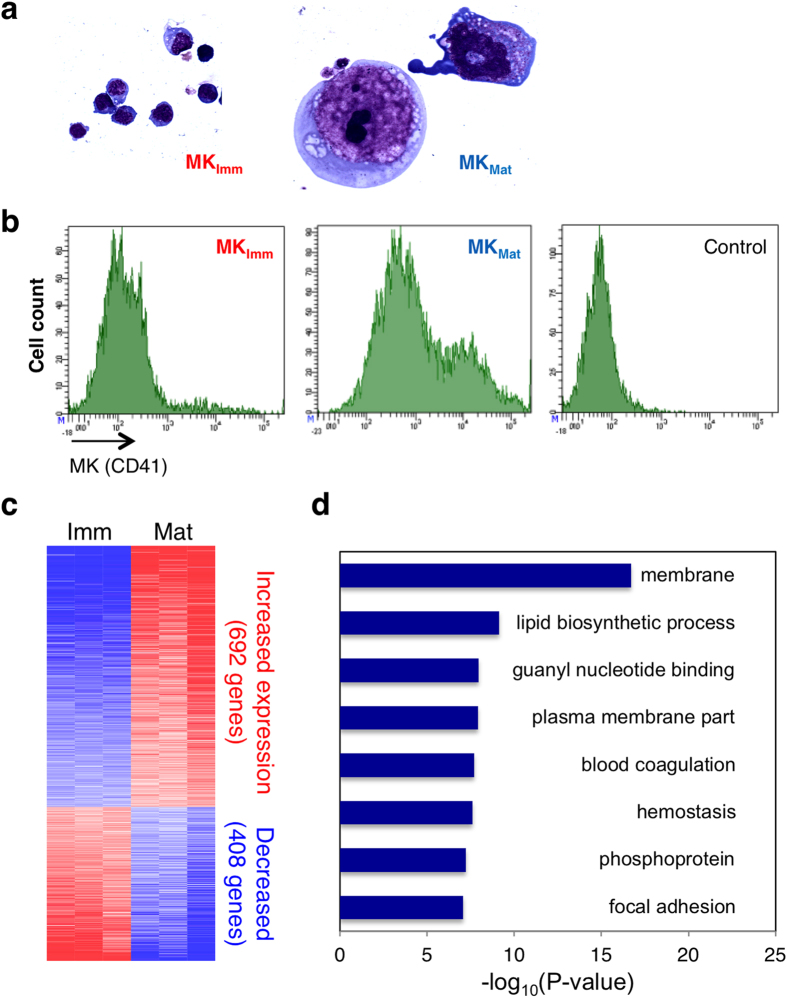
Megakaryocyte (MK) isolation and characteristics. (**a**) Representative mouse MK populations stained with May-Grünwald-Giemsa after immunomagnetic and density-gradient isolation 2 days (Imm) or 5 days (Mat) after culture of fetal liver cells in thrombopoietin. (**b**) Flow cytometry analysis of isolated MK_Imm_ and MK_Mat_. Morphology (A) and immunophenotype (B) together verify MK maturation and effective separation. (**c**) Heat map of genes differentially expressed in MK_Imm_ and MK_Mat_, as determined in triplicate Affymetrix microarrays (blue = low, red = high expression). This report centers on regulation of the 692 genes increased ≥1.5-fold (FDR <0.05) in MK_Mat_. (**d**) Gene Ontology analysis of genes expressed selectively in MK_mat_, showing high enrichment of functions classically attributed to mature MK and blood platelets.

To investigate chromatin and TF dynamics in MK maturation, we cultured mouse fetal liver cells in thrombopoietin (TPO), collecting young committed cells after 2 days of culture (MK_Imm_) and large, terminally mature cells at the peak of proplatelet formation 3 days later (MK_Mat_). We used Ly76 (Ter119), Ly6g (Gr1) and Itgam (CD11/Mac1) antibodies (Ab) to deplete other blood lineages and 3 sedimentations over bovine serum albumin to remove (in the case of MK_Imm_) or enrich (for MK_Mat_) large, mature cells. Histochemistry and flow cytometry verified the expected differences in cell populations (Fig. 1a,b). Moreover, Ly76^+^ erythrocytes were efficiently depleted (<2% residual) and small, unavoidable granulocyte/macrophage residuals were equal in both populations. RNA profiles confirmed lineage depletion and showed efficient separation of immature and mature MKs, revealing higher levels of 692 transcripts and lower levels of 408 genes in MK_Mat_ (Fig. 1c), on par with results from prior studies^27^. Gene Ontology analysis of the increased transcripts highlighted membrane-related function, guanyl nucleotide-regulated processes and hemostasis (Fig. 1d), processes known to dominate in terminal MK differentiation^27^,^28^.

**Figure 2:**
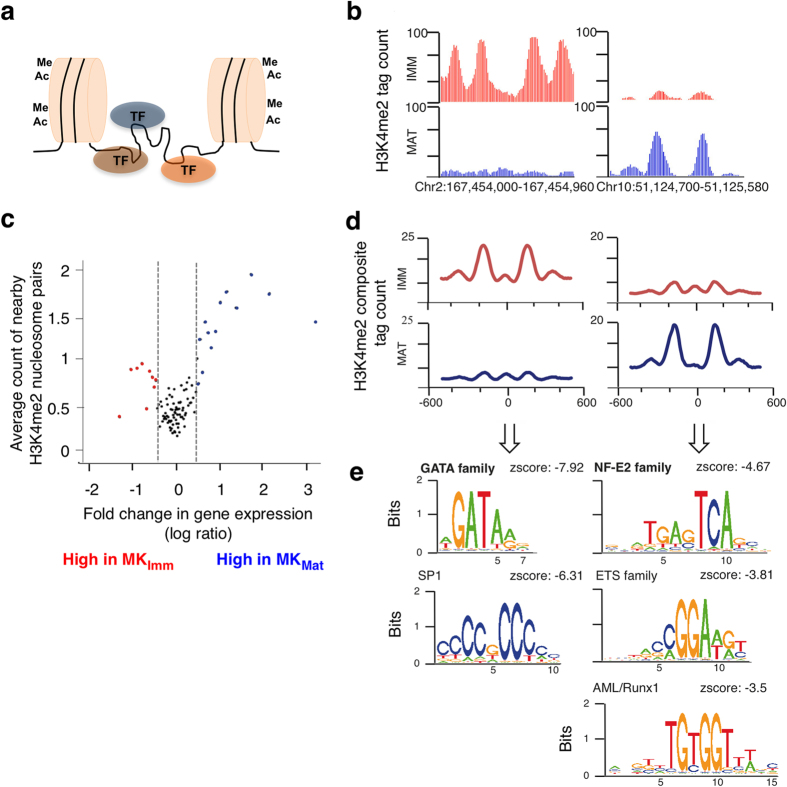
Analysis of H3K4me2-marked enhancers in immature and mature MK. (**a**) Diagram representing active enhancers, showing TF binding in nucleosome-depleted regions flanked by nucleosomes that carry active histone marks such as H3K4me2. (**b**) H3K4me2 ChIP-Seq data at two representative regions in MK_Imm_ (red) and MK_Mat_ (blue), illustrating differential enhancer activity in the two cell populations. (**c**) Differential gene expression (x-axis, bins of 50 genes each) plotted against average counts of H3K4me2-marked nucleosome pairs per bin. Dashed lines demarcate 1.5-fold differential mRNA expression. (**d**) Composite H3K4me2 tag counts from over 10,000 regions with differential chromatin structure in MK_Imm_ (red) and MK_Mat_ (blue), aligned at the center of nucleosome pairs. These plots represent the aggregate of signals such as those shown in B. (**e**) Transcription factor binding motifs significantly enriched (Z-score >3) near the troughs of paired nucleosomes represented in E. Similar motifs were merged and the one with highest Z-scores were selected as representative.

ChIP-seq for H3K4me2 on micrococal nuclease (MNase)-digested chromatin from the two MK populations identified thousands of H3K4me2-marked nucleosomes (Suppl. Fig. 1a). Many of these were present as nucleosome pairs separated by 250–450 bp of H3K4me2-depleted chromatin (Fig. 2a,b), a configuration that denotes active, TF-occupied *cis*-regulatory elements^21^,^22^. Nearly half of all such H3K4me2-marked nucleosome pairs localized at gene promoters (<2 kb from a transcription start site, TSS, Suppl. Fig. 1b) and showed little difference between immature and mature MK; this was expected because mammalian genes are controlled largely through distant enhancers^24^, including in MKs^26^. To investigate the relation of these paired nucleosomes with gene expression, we placed all expressed genes in bins of 50 genes each and determined the average number of nucleosome pairs located 2 kb to 20 kb from their TSSs. Differentially expressed genes showed more H3K4me2-marked nucleosome pairs than genes that express at similar levels in immature and mature MK (Fig. 2c). This association was particularly evident for genes activated in MK_Mat_ (blue dots), affirming that H3K4me2 ChIP at nucleosome resolution reveals functional enhancers.

To quantify chromatin dynamics at these sites, we assigned Nucleosome Stabilization-Destabilization (NSD) scores^21^,^22^, based on differences in H3K4me2 ChIP signals in MK_Mat_ and MK_Imm_. To identify TFs that may act selectively at enhancers in immature or mature MK, we searched for DNA sequence motifs enriched between all nucleosome pairs unique to each population (Fig. 2d). The GATA motif was enriched within sites in the highest percentile of MK_Imm_ selectivity (Fig. 2e), but not at sites enriched in MK_Mat_, consistent with known GATA1 requirements early in MK differentiation^20^,^29^,^30^. In contrast, the highest percentile of MK_Mat_-selective sites were enriched for sequences that bind NF-E2, ETS-family proteins, and RUNX1/AML (Fig. 2e), corresponding to three TFs - NF-E2, FLI1, and RUNX1 - whose absence arrests MK maturation, leading to thrombocytopenia *in vivo*^3^,^11^,^16^. Thus, a few TFs that bind hundreds of stage-restricted enhancers seem to control a large segment of the transcriptional program in late MK maturation.

### NF-E2 binds DNA at a high fraction of dynamic paired-nucleosome sites to activate MK genes

**Figure 3:**
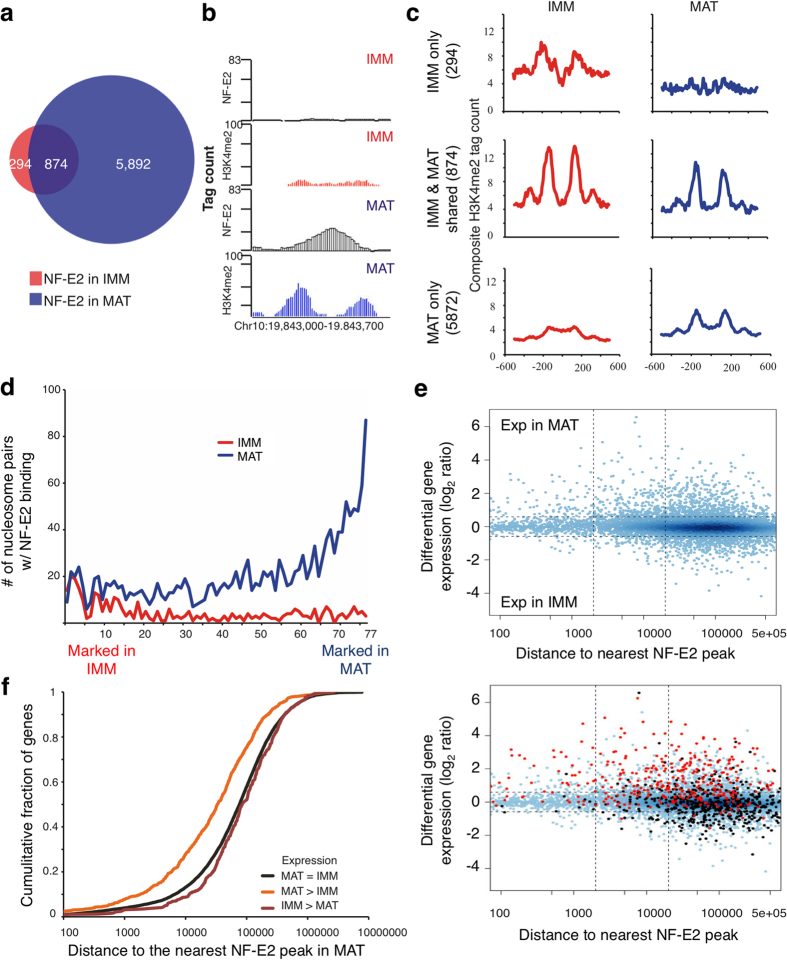
NF-E2 activates genes expressed late in MK maturation. (**a**) Overlap of NF-E2 binding sites in MK_Imm_ (red) and MK_Mat_ MAT (blue). (**b**) ChIP-seq signals for NF-E2 (black) and H3K4me2 (red and blue) at representative 1-kb regions in MK_Imm_ (red) and MK_Mat_ MAT (blue). (**c**) Composite H3K4me2 signals in MK_Imm_ (red) and MK_Mat_ MAT (blue) at NF-E2 binding sites present only in MK_Imm_ (top), sites common to both populations (middle row), and sites occupied only in MK_Mat_ (bottom). (**d**) NF-E2 shows a strong tendency to bind chromatin that is marked selectively in MK_Mat_. H3K4me2-marked nucleosome pairs (putative enhancers, x-axis) were ranked according to the degree of differential marking in MK_Imm_ (left) or MK_Mat_ (right) and grouped in bins of 1000 pairs. Y-axis represents the number of nucleosome pairs in each bin that show NF-E2 binding. (**e**) NF-E2 regulation map. Each dot represents a gene, with the x-axis marking the distance from its TSS to the nearest NF-E2 binding site in MK_Mat_ and the y-axis marking the log-scaled fold change in mRNA level during MK maturation. The horizontal lines demarcate 1.5-fold cutoffs for transcript levels expressed higher in MK_Imm_ (negative numbers) or higher in MK_Mat_ (positive numbers). Dots to the left of the first vertical line represent promoter binding (<2 kb from the TSS) and dots to the right represent binding >2 kb away. In the bottom panel, genes decreased (red) or increased (black) in *Nfe2*^−/−^, relative to wild-type, MK are projected on the above regulation map. Most genes diminished in the absence of NF-E2 (red dots) are expressed selectively in wild-type MK_Mat_. (**f**) Cumulative distribution of the distance from each gene’s TSS to its nearest NF-E2 binding site for genes expressed selectively in MK_Mat_ (orange), in MK_Imm_ (brown) or at similar levels in both stages (black). NF-E2 binds significantly closer to MK_Mat_-specific genes (*P* < 2.2 × 10^−16^ by the K-S test) and further from MK_Imm_ (*P* = 0.03) genes, compared to the background for invariant genes.

Mice lacking NF-E2, the factor with the most enriched motif in areas of dynamic chromatin in MK_Mat_, are profoundly thrombocytopenic^11^,^13^. To determine NF-E2 interactions with stage-specific chromatin and gene regulation, we used p45 NF-E2 Ab for ChIP-seq (Suppl. Fig. 1a), identifying few binding sites unique to MK_Imm_ and thousands of sites exclusive to MK_Mat_ (Fig. 3a). The number of confident binding sites was considerably higher than identified in a previous study^31^ and the canonical NF-E2 motif 32,33 was the most enriched (Z-score = −135.2) at these sites, which lay mainly in introns or intergenic regions >2 kb from TSSs and showed high phylogenetic conservation (Suppl. Fig. 1c). Individual (e.g., Fig. 3b) and aggregate (Fig. 3c) profiles revealed significant binding in selective regions with H3K4me2-marked nucleosome pairs. To examine this relationship further, we arranged all ~77,000 non-promoter nucleosome pairs into bins of 1,000, ranked by the NSD score, i.e., the magnitude of difference in H3K4me2 signal between MK_Mat_ and MK_Imm_ (Fig. 3d, x-axis). Most of the top-ranked bins showed frequent NF-E2 binding, which was evident in 5% to 9% of the 5,000 highest-scoring regions (Fig. 3d). Together with the large number of binding sites unique to MK_Mat_, these observations reveal the scope of potential NF-E2 function at enhancers that are active late in MK maturation.

To determine if this binding is functional, we first examined NF-E2 occupancy near genes that are selectively active in MK_Mat_. To this end, we represented differential expression of all genes in MK_Imm_ and MK_Mat_ on a scatter plot in relation to the distance from the TSS to the nearest NF-E2 binding site (Fig. 3e top). This illustrated that NF-E2 binds significantly closer to genes with high expression in MK_Mat_ than to invariant genes or those with high expression in MK_Imm_ (also shown in Fig. 3f, *P* < 2.2 × 10^−16^ by the KS test). Beyond a distance of 20 kb, similar numbers of genes fall above and below the horizontal lines in Fig. 3e, which indicates that about as many genes with distant NF-E2 binding increase as decrease expression in MK_Mat_. Accordingly, for the subsequent purpose of defining enhancers, we consider 20 kb an empiric boundary for a large fraction of functional binding sites.

To assess gene dependence on NF-E2, we projected gene expression changes identified in *Nfe2*^−/−^ MKs^34^,^35^, which were cultured and harvested similarly to our procedures^34^, on the scatter plot, marking genes that decrease in *Nfe2*^−/−^ MKs, compared to wild-type cells, in red and those that increase in black (Fig. 3e bottom). We observed higher NF-E2 occupancy near genes with reduced transcript levels in the mutant cells, compared to those that increase or stay fixed (*P* < 2.2 × 10^−16^). Although transcript levels in *Nfe2*^−/−^ MKs again revealed 20 kb as the distance to impute enhancers with confidence, many of these NF-E2-dependent genes showed binding at even larger distances (Fig. 3e bottom). Thus, NF-E2 activates genes, with little direct effect on gene silencing, mainly through enhancers. Transcripts reduced in *Nfe2*^−/−^ MKs could be direct transcriptional targets or merely reflect arrested cell maturation. Among reported candidate target genes^27^,^36^,^37^,^38^,^39^, *Tubb1*, which is highly expressed in wild-type MK_Mat_ and absent in *Nfe2*^−/−^ MKs^40^, showed no NF-E2 occupancy within 250 kb (Suppl. Fig. 1d). In contrast, *Tbxas1*, *Casp12*, *Lims12* and *Rab27b* showed nearby NF-E2 binding (Suppl. Fig. 1e and data not shown). Moreover, NF-E2 bound DNA within 20 kb of 270 out of 692 highly MK_Mat_-selective genes (39%, Suppl. Table 1), compared to 60 of 408 MK_Imm_-specific genes (14.7%, *P* = 1.3 × 10^−18^ by Fisher’s exact test). Together, these data confirm the scope of NF-E2 activity and can explain the profound maturation arrest in *Nfe2*^−/−^ MKs.

### Roles for RUNX1 and FLI1 in MK maturation

**Figure 4:**
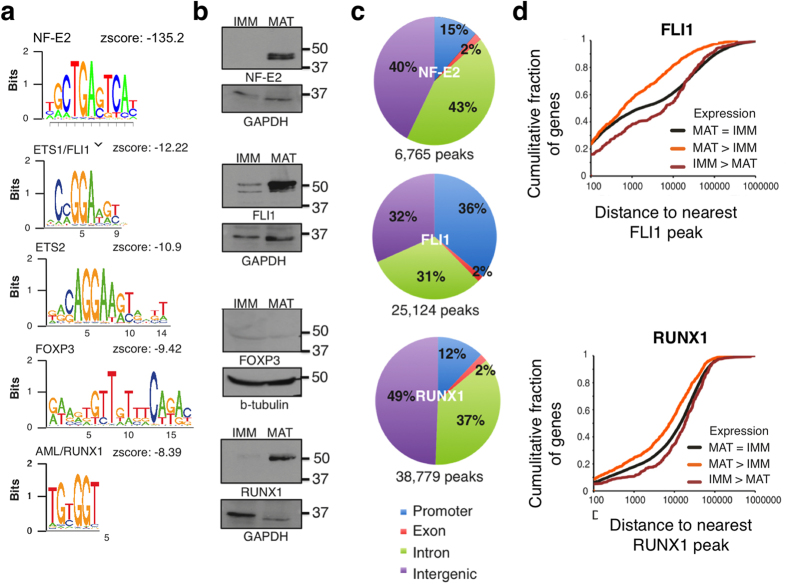
Expression and binding of TFs with highly enriched recognition motifs detected near NF-E2 binding sites. (**a**) Sequence motifs most enriched near sites of NF-E2 occupancy in MK_Mat_. (**b**) Immunoblots showing high levels of NF-E2, FLI1 and RUNX1 in MK_Mat_ compared to MK_Imm_. FoxP3 was not detected and GAPDH or TUBB served as loading controls. **(c)** Genomic distributions of NF-E2, FLI1 and RUNX1 binding sites in MK_Mat_. (**d,e**) Cumulative distribution of the distance from each gene’s TSS to its nearest FLI1 (**d**) or RUNX1 (**e**) binding sites for different gene sets. Both TFs bind significantly closer to MK_Mat_ genes (orange; *P* < 2.2 × 10^−16^ for FLI1, *P* = 2.28 × 10^−13^ for RUNX1 by the K-S test) compared to invariant genes and further from MK_Imm_ genes (brown; *P* = 1.08 × 10^−5^ for FLI1, *P* = 1.67 × 10^−3^ for RUNX1).

To identify TFs that may collaborate in this activity, we searched for sequence motifs enriched near NF-E2 binding sites in mature MK. Motifs corresponding to NF-E2, ETS proteins (such as ETS1 and FLI1), FOXP3, and AML/RUNX1 were the most highly represented (Fig. 4a); notably, three of these motifs were also enriched at sites of dynamic chromatin in MK_Mat_ (Fig. 2e). Neither immunoblotting (Fig. 4c) nor qRT-PCR (data not shown) detected FOXP3 in mouse MKs, suggesting that some other TF may bind that motif. Moreover, among ETS factors, *Ets1* and *Ets2* mRNA levels fall during MK maturation (Suppl. Fig. 2a) and *Ets1*^−/−^ or *Ets2*^−/−^ mice lack MK or platelet defects^41^. In contrast, levels of another ETS protein, FLI1, rise significantly in MK_Mat_, similar to p45 NF-E2 (Fig. 4b), and *Fli1*^−/−^ mice show profound dysmegakaryopoiesis^14^,^15^. Moreover, RUNX1 and FLI1 commonly co-occupy DNA in human and mouse MKs^19^,^20^ and in HPC-7 cells^18^. NF-E2 binding in MK_Mat_ also occurred at many sites that bind FLI1 and RUNX1 in HPC-7 cells (Suppl. Fig. 2b) and NF-E2 binds the *Fli1* and *Runx1* loci in wild-type MK_Mat_ (Suppl. Fig. 2c), implying a TF network, a common feature of stable differentiated cells^42^. These observations collectively suggest that NF-E2, FLI1 and RUNX1 are key transcriptional regulators of terminal MK maturation.

ChIP-seq for FLI1 and RUNX1 revealed thousands of confident binding sites for the two TFs (Suppl. Fig. 1a) and encompassing nearly all previously mapped RUNX1 binding sites^43^. Motifs corresponding to each TF were the most significantly enriched in the respective ChIP fragments (Z-score −143.9 for FLI1, −11.7 for RUNX1), which implies their direct binding to DNA. Both factors bound mainly in intergenic regions and introns, similar to NF-E2, although about 1/3 of FLI1 binding occurred at promoters (Fig. 4c). Also resembling NF-E2, both FLI1 and RUNX1 tend to bind closer to genes that increase expression in MK_Mat_ and further from MK_Imm_ genes (Fig. 4d), suggesting that all three TFs activate genes in maturing MK. Coupled with stage-selective *cis*-regulatory regions, the binding profiles of 3 abundant, essential TFs provided information relevant to the transcriptional basis of MK maturation.

**Figure 5:**
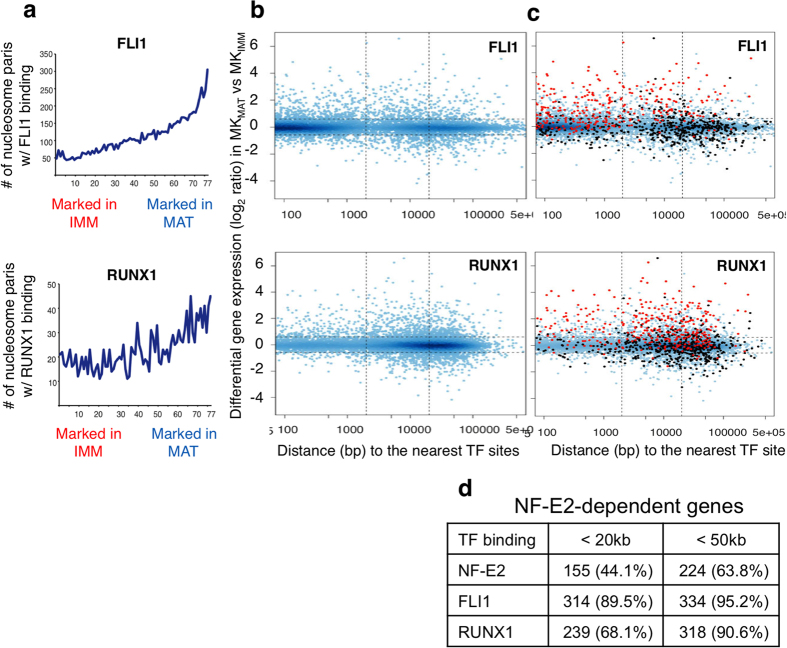
Features of FLI1 and RUNX1 binding in terminally mature MK. (**a**) The tendency of FLI1 (top) and RUNX1 (bottom) to bind at H3K4me2-marked, open chromatin in MK_Mat_. The 77,000 nucleosome pairs identified in MK (Fig. 2) are binned in groups of 1,000 (x-axis, as described for Fig. 3d) and the number of nucleosome pairs having FLI1 or RUNX1 binding sites in each bin is plotted on the y-axis. (**b**) Regulation maps of FLI1 (top) and RUNX1 (bottom), prepared as described for NF-E2 in Fig. 3f. Each dot represents a gene, with the x-axis marking the distance from its TSS to the nearest TF binding site and the y-axis marking the log-scaled fold-change in transcript level between MK_Mat_ and MK_Imm_. (**c**) Genes with reduced (red dots) or elevated (black dots) expression in *Nfe2*^−/−^, compared to wild-type, MK^35^ are projected onto these regulation maps to survey potential roles for FLI1 and RUNX1 in regulating NF-E2-dependent genes. (**d**) Frequency of TF binding sites within 20 and 50 kb of the TSSs of NF-E2-dependent genes (reduced expression in *Nfe2*^−/−^ MK).

To investigate this basis, next we mapped FLI1 and RUNX1 occupancy at differentially active regulatory regions, i.e., in relation to NSD scores (differences in H3K4me2 marking). RUNX1 binding was modestly enriched among enhancers selectively marked in MK_Mat_, whereas FLI1 binding was evident at 15% to 30% of enhancers with the greatest differential marking (Fig. 5a). The extent of FLI1 binding reflects the 4-fold excess of FLI1 over NF-E2 binding sites and suggests that FLI1 may control an especially large fraction of late MK genes. We therefore considered every gene with respect to its differential expression in MK_Mat_ *vs.* MK_Imm_ (y-axis in Fig. 5b) and the nearest binding of each TF (x-axis). This analysis revealed first that each TF binds DNA mainly near genes that increase expression significantly in MK_Mat_ (Fig. 5b), similar to NF-E2; this is especially the case at distances under 20 kb. Second, RUNX1 and particularly FLI1 bind the promoters of many more MK_Mat_ genes than does NF-E2, although, both TFs occupy many more distant sites than promoters, consistent with the bulk of gene regulation occurring at enhancers.

To impute the functions of FLI1 and RUNX1 binding with respect to NF-E2, we examined each TF’s occupancy near genes that are enriched in MK_Mat_ and depend on NF-E2 *in vivo*^34^. Figure 5c projects these genes in red (reduced expression in *Nfe2*^−/−^ MK) or black (levels rise in *Nfe2*^−/−^ MK) onto the adjoining (Fig. 5b) regulation maps. The densities of red dots in each frame reveal extensive FLI1 and RUNX1 binding near NF-E2-dependent genes, and in contrast to the few such genes with promoter (<2 kb) binding of NF-E2 or RUNX1, nearly 2/3 of these promoters showed FLI1 occupancy (Fig. 5c). FLI1 also bound about half as many enhancers as RUNX1 or NF-E2. Notably, whereas 44.1% of NF-E2-dependent genes bind NF-E2 within 20 kb and 63.8% of these genes bind NF-E2 within 50 kb, the fraction of genes that bind within 20 kb is higher for both RUNX1 (68.1%) and FLI1 (89.5%) binding (Fig. 5d). These associations indicate extensive co-regulation of MK_Mat_ genes by these three TFs.

### Singular and combinatorial TF activity in MK maturation

**Figure 6:**
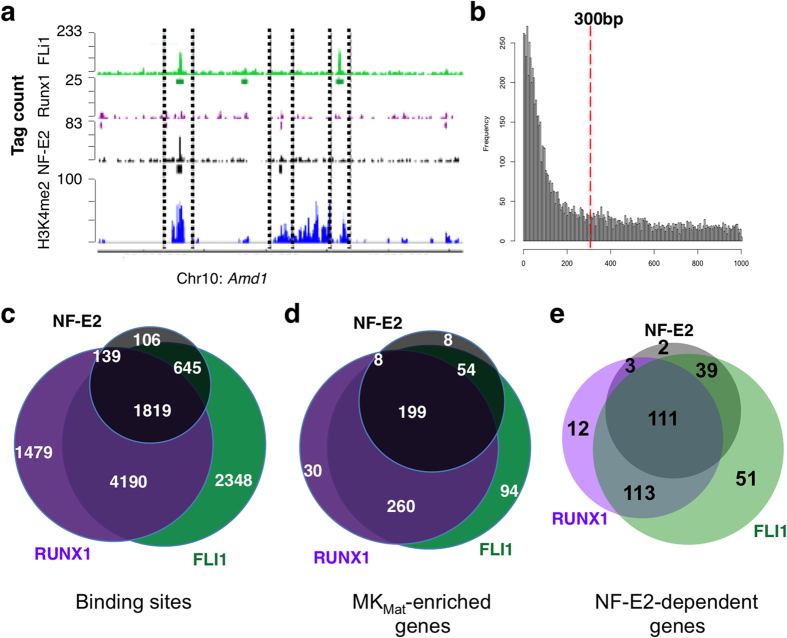
Combinatorial TF activity in mature MK. (**a**) ChIP-seq data traces at a representative MK_Mat_-specific locus, *Amd1*, showing permutations of solitary and combined TF occupancy and H3K4me2 marks within pairs of dotted lines. **(b)** Histogram of the distances between binding summits for NF-E2, FLI1 and RUNX1, showing the empiric basis for our choice of 300 bp as the criterion for TF co-occupancy. (**c–e**) Venn diagrams showing the overlap of *all* FLI1, RUNX1 and/or NF-E2 *binding sites* (**c**); the TF binding at the enhancer regions of the *MK*_*Mat*_*-selective genes* that bind any of the three TFs (**d**); and the co-binding of the 3 TFs at genes that *depend on NF-E2 in vivo* (**e**).

As it is increasingly clear that multiple *cis*-elements control individual genes, NF-E2, FLI1 and RUNX1 could co-regulate MK_Mat_ genes through binding at the same or different enhancers. This is an important distinction because 2 of these 3 TFs co-occupy DNA in other contexts^18^,^19^, albeit without clear functional consequence. Moreover, the individual profiles for FLI1, RUNX1 and NF-E2 revealed all possible binding combinations: alone, in pairs, or as a trio (Fig. 6a). We therefore sought to determine the prevalence and roles of solitary and combined TF binding.

By surveying the interval distances between summits of all ChIP-seq peaks for the 3 TFs, we identified 300 bp as the upper limit that likely encompasses discrete regulatory elements (Fig. 6b). With this parameter, although each TF showed some binding without concomitant binding of another, we detected substantial co-occupancy, ranging from 74% of all FLI1 binding sites to 96% of all NF-E2 binding sites; more than 1,800 regions bound all three TFs and few regions bound only NF-E2 and RUNX1 (Fig. 6c). Considering the 692 genes expressed selectively in MK_Mat_, we observed TF co-occupancy in the enhancers of 84.5% of all FLI1-bound genes and 94% of all RUNX1-bound genes; indeed, only 8 of 269 NF-E2-bound regions lacked another TF (Fig. 6d). Finally, near genes that depend on NF-E2 *in vivo*^34^, we detected substantial co-occupancy with at least FLI1 or RUNX1 and commonly both (Fig. 6e). Thus, co-occupancy of TFs essential to MK function is pervasive and particularly evident in the vicinity of MK_Mat_-selective genes. In particular, genes frequently bind FLI1 at their promoters and show significant binding of NF-E2 and FLI1, with or without RUNX1, at distant enhancers.

**Figure 7:**
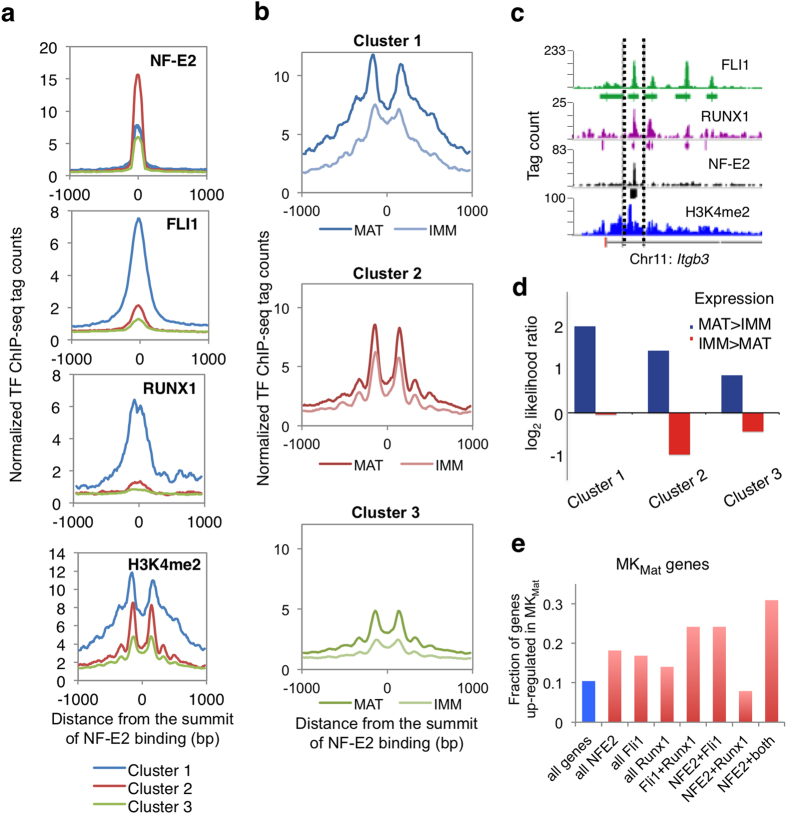
Functional analysis of TF co-occupancy at MK enhancers. (**a**) Composite ChIP-seq signals for NF-E2, FLI1, RUNX1, and H3K4me2 at 3 distinct clusters of NF-E2 binding sites, identified by K-means clustering of binding sites for the three TFs and described in the text. (**b**) Composite ChIP-seq signals for H3K4me2 in MK_Imm_ and MK_Mat_ at the 3 clusters of NF-E2 binding sites. (**c**) ChIP-seq data traces at a representative MK_Mat_-specific locus, *Itgb3*, showing all three TFs occupying a putative regulatory region marked with H3K4me2. Other sites in the locus show binding of one or two TFs. (**d**) Odds ratios of genes near (<20 kb) NF-E2 binding sites in each cluster showing higher expression in MK_Mat_ (blue) or in MK_Imm_ (red), relative to the genome background. (**e**) Proportion of genes with nearby binding of the various combinations of NF-E2, FLI1, and RUNX1 among genes showing higher expression in MK_Mat_. The data indicate the functional role of FLI1, in conjunction with NF-E2 or RUNX1 and especially with both TFs, in regulating genes expressed in mature, but not in immature (Suppl. Fig. 2d) MK.

To ask how combinations of NF-E2 with other TFs might affect MK gene expression, we used *k*-means clustering, an unbiased and unsupervised method, to sub-divide NF-E2 binding sites into 3 clusters based on the ChIP-seq signal and FLI1/RUNX1 co-occupancy (Fig. 7a). H3K4me2 levels in all 3 clusters of NF-E2 binding sites were increased from MK_Imm_ to MK_Mat_ and enhancers with high FLI1 and RUNX1 occupancy (cluster 1) showed the largest average gain in H3K4me2 during MK maturation (Fig. 7b). Among all NF-E2-bound enhancers, cluster 1 also showed the highest H3K4me2 signals in flanking nucleosomes (Fig. 7a, blue curves). These features are nicely illustrated at the *Itgb3* locus, which is strongly activated during MK maturation and binds each TF in close proximity within H3K4me2-marked intronic sites (Fig. 7c). NF-E2 binding signals were highest, and H3K4me2 signals remained robust, when only one of the other TFs was also present (cluster 2, where average signals from RUNX1 and FLI1 binding were also considerably lower); H3K4me2 signals were the lowest when NF-E2 was found alone (cluster 3) (Fig. 7a). Notably, enhancers that bound 2 or 3 TFs were associated with a significantly higher probability of selective expression of nearby genes in MK_Mat_. This relationship was most clear and extreme for sites that bind all three TFs (cluster 1) but was also evident for sites that bind just one TF in addition to NF-E2 (Fig. 7d). Thus, enhancers that bind 2 or all 3 TFs mark MK_Mat_-selective genes best.

Because the foregoing analysis centered on *binding sites*, next we considered TF occupancy with respect to the 692 *genes* expressed selectively in MK_Mat_ (Suppl. Table 1). Relative to the background for all genes, those with nearby binding of NF-E2, FLI1 or RUNX1 were enriched for MK_Mat_-specific genes (Fig. 7e). Co-occupancy of FLI1 with NF-E2 or RUNX1 showed even better association with MK_Mat_-specific genes, implying that FLI1 collaborates productively with both TFs. Importantly, nearly 1/3 of genes that bind all 3 TFs in close vicinity are MK_Mat_-selective genes (right-most bar in Fig. 7e), indicating potent additive effects. This association is unlikely to be spurious because pairing of NF-E2 and RUNX1 is much less frequent, suggesting that the latter TF pair may have a limited regulatory role in the absence of FLI1. Furthermore, near genes that bind any of the three TFs alone or in combination, we observed no enrichment - and even some depletion - of genes expressed selectively in MK_Imm_ (Suppl. Fig. 2d).

## Discussion

Much of the current appreciation of the transcriptional control of thrombopoiesis rests on findings in knockout mice and in human pedigrees with syndromic thrombocytopenia, which point separately to three necessary TFs – NF-E2, FLI1 and RUNX1. It is, however, unclear if these are the principal transcriptional determinants of platelet assembly, as other TFs may be equally essential. Moreover, although NF-E2 is activated late in MK maturation and defects in *Nfe2* mutant mice are confined to the final steps in platelet assembly, FLI1 and RUNX1 also show activities in young MKs; thus, defective thrombopoiesis in the absence of the latter factors might reflect those early roles or additional functions in terminally mature cells. The basis for the strong functional overlap among TFs with distinct DNA-binding preferences is also unclear, particularly if they act at promoters or enhancers, and collaborate at the same late-active MK genes or regulate different transcriptional targets. We therefore studied chromatin dynamics during MK differentiation and accurately mapped TF occupancy in terminally mature MK. Our application of the genome-wide histone mark H3K4me2 in different cell states to identify TF activity highlights the rich information contained in dynamic chromatin and the power of this information to reveal gene regulatory mechanisms in primary cells.

In MKs and other cells, histone modifications are much more dynamic at enhancers than at promoters^24^,^26^. The hundreds of distant *cis*-elements that acquire hallmarks of activation in terminally mature MKs hence represent the principal sites of relevant TF activity, and sequence motifs corresponding to NF-E2, FLI1 and RUNX1 are by far the most enriched within these regions. FLI1 and RUNX1 were also the next most enriched motifs (after NF-E2) at sites of NF-E2 binding and no other DNA sequences were significantly enriched at regions of FLI1 and RUNX1 occupancy. Although various TFs can in principle bind the same motifs, NF-E2, FLI1 and RUNX1 are the dominant family members expressed selectively in MKs and notably increased in terminal cells. When considered in the light of the thrombopoietic defects associated with mutations in each of these genes, our observations imply that NF-E2, FLI1 and RUNX1 together control much of the late MK transcriptional program, though additional TFs – such as one that binds the Fox motif enriched near NF-E2 binding sites – likely have supporting roles. Most of the ~700 genes activated late in MK maturation show nearby binding of at least one – and often 2 or all 3 – of these TFs, mainly in regions that carry the activated histone mark H3K4me2 only in mature cells. Although gains and losses in many transcripts accompany MK differentiation, genes that are selectively active in terminally mature cells show far greater binding than genes that are silenced, which indicates a predominant activating role for NF-E2, FLI1 and RUNX1, with probably little to no activity in transcriptional repression.

These features contrast with those of other TF families – GATA1/2 and TAL1/LYL1 – that are also expressed in erythroid, MK, and other blood cells. Elegant studies have highlighted activities of the latter TFs, which mainly function early in MK differentiation and activate or repress transcription, depending on the cellular context and associated protein complexes^19^,^20^,^44^,^45^. Moreover, in progenitor cells GATA and TAL proteins seem to prime enhancers for subsequent activity in specified MKs, probably in conjunction with RUNX1 and FLI1^19^,^20^, which are present at low levels in mouse MK_Imm_ (Fig. 4b). Indeed, WGATAR was the most enriched motif in enhancers that are selectively active in immature MK, consistent with known GATA1 requirements in these cells^29^. NF-E2, FLI1 and RUNX1 levels increase dramatically in terminal MKs and our study highlights their occupancy at newly activated enhancers.

The frequent association of NF-E2, FLI1 and RUNX1 in various combinations at or near late-MK genes is of particular note. Histones showed low H3K4me2 at enhancers occupied by single TFs and the highest levels of this activation mark in areas that bound NF-E2 and either RUNX1, FLI1 or both TFs. Co-occupied enhancers were also the best associated with genes expressed selectively in MK_Mat_ and gave the strongest signals for TF occupancy. Taken together, these findings reveal the importance of these TFs at late-MK enhancers and provide a basis to understand why their absence compromises thrombopoiesis. Notably, loss of any member of this triad produces related cellular defects, each associated with thrombocytopenia. In particular, nearby binding of RUNX1 and/or FLI1, which is evident at most genes with reduced expression in *Nfe2*-null mice (Fig. 5c,d), seems insufficient to drive transcription. Thus, although various combinations of these TFs co-regulate many of the same target genes through discrete enhancers, their functions are not overtly redundant but complementary and individually essential.

Finally, our ChIP-seq data from primary cells will serve as a vital community resource to study epigenome regulation of platelet biogenesis. Genomic data revealing the collaborative functions of three TFs – NF-E2, FLI1 and RUNX1 – provide a foundation to uncover detailed mechanism their collaboration at dynamic enhancers to activate MK-selective genes. Those mechanisms will in turn lead to refined insights into how platelet biogenesis may be manipulated to manage disorders of platelet deficiency or excess. Although our conclusions are based on MKs cultured from mouse fetal livers, and human or adult mouse MKs may well show some differences, the overall regulatory logic of combinatorial TF activity at MK enhancers is probably conserved.

## Methods

### Cell culture and verification

Fetal livers from the CD1 strain of mice were collected on embryonic day 14 (E14) and single-cell suspensions were prepared by filtration through a 40-μm cell strainer, followed by successive passing through 18- to 23-gauge needles. All methods were carried out in accordance with guidelines established by the Animal Care and Use Committee of the Dana-Farber Cancer Institute and all experimental protocols were approved by this committee. After removal of erythrocytes in ammonium chloride-potassium (ACK) lysis buffer, cells were cultured in Dulbecco’s Modified Eagle Medium (Invitrogen) supplemented with 10% fetal bovine serum (FBS) and thrombopoietin (TPO, 1% culture supernatant from a producer cell line^46^). After 2 or 5 days of culture, cells were subjected to negative selection with TER119, GR1 and CD11b antibodies (Ab, BD Pharmingen; catalog #553671, 553123 and 553308, respectively) and magnetic Dynabeads (Invitrogen, catalog #110.35), followed by positive (MK_Mat_) or negative (MK_Imm_) selection over bovine serum albumin (BSA: 4%, 3%, 1.5%) gradients. May-Grunwald Giemsa staining of cytocentrifuged cells from each culture verified proper isolation. MK cultured for 2 or 5 days were also stained with FITC-labeled CD41, APC-labeled TER119, APC-labeled GR1 or PE-labeled CD11b Ab (1:200, BD Pharmingen; catalog #553848, 557909, 553129 and 557397, respectively) for 20 min at 4 °C, washed in cold phosphate-buffered saline (PBS) containing 2% FBS, incubated in Hoechst dye (1:10,000), and analyzed on a FACSCanto II flow cytometer (BD Biosciences).

### Expression, gene association, and Gene Ontology (GO) analyses

RNA isolated from purified MK using RNeasy Mini kits (Qiagen, catalog# 74104) was processed and hybridized to Mouse Genome 430A 2.0 microarrays (Affymetrix) according to the manufacturer’s instructions. Microarray experiments were done in triplicate. Data were processed using robust multiarray analysis (RMA) to normalize expression indices^47^. Genes with a unique RefSeq ID assigned to the probe set and called as “present” in at least 1 sample were retained for analyses. Differentially expressed genes between MK_Imm_ and MK_Mat_ were identified using LIMMA^48^, with false discovery rate (FDR) <0.05 and fold-change ≥1.5. GO analysis was performed using DAVID tools^49^ with default parameters, and GO terms with FDR <0.001 were selected. cDNA for RT-PCR analysis was synthesized using the QuantiTect reverse transcription kit (Qiagen, Catalog# 205311). Down- or up-regulated genes in *Nfe2*^−/−^ MK^34^ were chosen on the basis of ≥2-fold change on the microarray expression indices between wild-type and *Nfe2*^−/−^ cells.

### Immunoblotting

Cells were lysed in RIPA buffer and boiled in Laemmli sample buffer for 5 min before fractionation by 10% SDS-PAGE. After transfer to nitrocellulose membranes over 1.5 h at 65 V, blots were blocked with 5% milk in PBS containing 0.1% Tween-20 (pH 7.5) and incubated with p45 NF-E2 (1:1000, ref. 39.), FOXP3 (eBioscience, catalog #14-5773), RUNX1 (Abcam, catalog #23980), or FLI1 (Abcam, catalog #15289) Ab overnight at 4 °C. Blots were washed in PBS-Tween, incubated with horseradish peroxidase-conjugated goat anti-rabbit IgG (Santa Cruz, catalog #sc-2054, 1:2,000), and exposed briefly to chemiluminescence reagents (Santa Cruz, catalog #sc-2048).

### ChIP-seq for H3K4me2 at nucleosome resolution and for TFs

ChIP on purified MK was performed as described previously^22^, with H3K4me2 Ab (Millipore, catalog #07-030) and input chromatin control following chromatin digestion with micrococcal nuclease, or with Ab against p45 NF-E2^39^, RUNX1 (Abcam, catalog #23980) or FLI1 (Abcam, catalog #15289) after chromatin was sheared by sonication. Libraries, prepared using ThruPlex-FD kits (Rubicon Genomics) and at least 30 ng DNA from 3 or more precipitates was pooled for sequencing on an Illumina Hi-Seq instrument.

### ChIP-seq data analysis

ChIP-seq reads were mapped to mouse genome build mm9 using Bowtie with default parameters, and uniquely mapped, non-redundant reads were retained^50^. H3K4me2 ChIP-seq reads from MK_Imm_ and MK_Mat_ were merged, and H3K4me2-marked nucleosomes were identified using NPS^51^ with default parameters from the merged ChIP-seq library. H3K4me2-marked nucleosome pairs, NSD scores during MK maturation, and sequence motifs enriched among the most dynamic nucleosome pairs were identified using BINOCh^52^ with default parameters. NF-E2, FLI1, and RUNX1 binding sites in the genome were identified using MACS 1.4 with default parameters^53^. Motifs enriched at TF binding sites were identified using SeqPos^54^ on the Cistrome analysis platform^55^. Whereas all reported sequence motifs have a *P*-value less than 0.001, SeqPos uses a Z-score to measure the enrichment level of a motif based on both frequency of occurrence and proximity to the peak summits (Figs 2e and 4a). ChIP-seq data were represented in wiggle format generated from NPS for H3K4me2 or from MACS for TFs, respectively, and were visualized using IGV Genome Browser (Figs 2b, 3b, 6a and 7c).

### Composite profile of ChIP-seq signal density on a set of regions

Anchored regions were aligned by the summit locations for TF binding sites (Figs 3c and 7a,b) or center locations for nucleosome pairs (Fig. 2d), and ChIP-seq reads were tallied in non-overlapping 10-bp windows. The genomic location of a sequence read was shifted in the 3′ direction by half the average ChIP DNA fragment size to represent the center of the relevant fragment, estimated by calculating the cross-correlation between all 5′ and 3′ reads^50^ (i.e., 150 bp for most datasets). Total read counts were then normalized to RPKM by the total non-redundant read count for each dataset.

### Integrative bioinformatics analyses

The summit of the MACS-identified ChIP-seq peak was considered as the location of a TF binding site. In TF regulation maps (Figs 3e and 5b,c), the distance from a gene to its nearest TF binding site is calculated by the distance on chromosomal locations between the TSS of that gene and the summit of the nearest peak, upstream or downstream, regardless of the presence of intervening genes, if any. Co-occupancies of multiple TFs were determined under the criterion that the distance between the summits of any two binding sites was lower than the inflection point in the distribution of such distances, i.e., 300 bp (Fig. 6b). *K*-means clustering of NF-E2 binding sites based on FLI1 and RUNX1 co-occupancy (Fig. 7a,b) was performed on the normalized wiggle profiles of NF-E2, FLI1, and RUNX1 ChIP-seq signal extracted from a 1-kb region centered at each NF-E2 peak summits, using the Heatmap tool on the Cistrome analysis platform. Among the different values we tested, *k* = 3 gave simple and the clearest separation based on ChIP-seq signal patterns. ChIP-seq and RNA expression profiling microarray data are deposited in the Gene Expression Omnibus (accession numbers GSE42108 and GSE42110).

## Additional Information

**How to cite this article**: Zang, C. *et al.* NF-E2, FLI1 and RUNX1 collaborate at areas of dynamic chromatin to activate transcription in mature mouse megakaryocytes. *Sci. Rep.* **6**, 30255; doi: 10.1038/srep30255 (2016).

## Supplementary Material

Supplementary Information
