# Supplementary material for: NF-E2, FLI1 and RUNX1 collaborate at areas of dynamic chromatin to activate transcription in mature mouse megakaryocytes
Source: Sci Rep. 2016 Jul 26;6:30255. doi: 10.1038/srep30255 (PMC4960521; doi:10.1038/srep30255)
Supplement: Supplementary Information [file srep30255-s1.pdf]

NF-E2, FLI1 and RUNX1 collaborate at areas of dynamic chromatin to activate transcription in mature mouse megakaryocytes

Chongzhi Zang, **Annouck Luyten**, **Justina Chen**, **X. Shirley Liu**, **and Ramesh A. Shivdasani**

SUPPLEMENTARY MATERIALS

Supplementary Figure and Table Legends

Suppl. Figure 1. ChIP-seq analysis of NF-E2 and other megakaryocyte TFs. (a) Summary of unique sequence tags and peak calls from ChIP-seq experiments with different antibodies in MK cell populations. **(b)** Distribution of H3K4me2 marked nucleosomes in MK, showing that the majority lie in introns and intergenic regions. **(c)** PhastCons conservation plot of NF-E2 binding sites. (**d,e)** Integrated Genome Viewer (IGV) traces showing NF-E2 occupancy far (>250 kb) from *Tubb1* (d) and at 5 sites within 20 kb of *Tbxas1* (e). Magnified regions on the right highlight NF-E2 binding in H3K4me2-marked chromatin.

Suppl. Figure 2. Features of FLI1 and RUNX1 expression and binding in MK. (a) mRNA expression levels of TF genes in immature (IMM) and mature (MAT) MK, as determined on Affymetrix microarrays. **(b)** IGV traces showing co-localization of FLI1 and RUNX1 in HPC-7 cells (top 2 rows) and binding of NF-E2 within H3K4me2-marked chromatin at some of the same sites in mature MK (bottom 4 rows). **(c)** IGV traces showing NF-E2 occupancy at the *Fli1* (top) and *Runx1* (bottom) gene loci. **(d)** Proportion of genes with nearby binding of the various combinations of NF-E2, FLI1, and RUNX1 that show higher expression in MK_{Imm}, relative to the genome background.

Suppl. Table 1. Differential expression and TF regulation pattern of 692 genes that were up-regulated during MK maturation.

Suppl. Figure 1

A

Antibody used for ChIP	Megakaryocyte population	Uniquely mapped ChIP-seq tags	MACS peaks	Positioned nucleosomes
H3K4me2	IMM	29,513,054	48,279	242,533
H3K4me2	MAT	16,032,555	68,115	246,089
NF-E2	IMM	24,887,829	1,168	
NF-E2	MAT	25,310,749	6,765	
FLI1	MAT	71,361,159	25,124	
RUNX1	MAT	45,311,238	38,779	

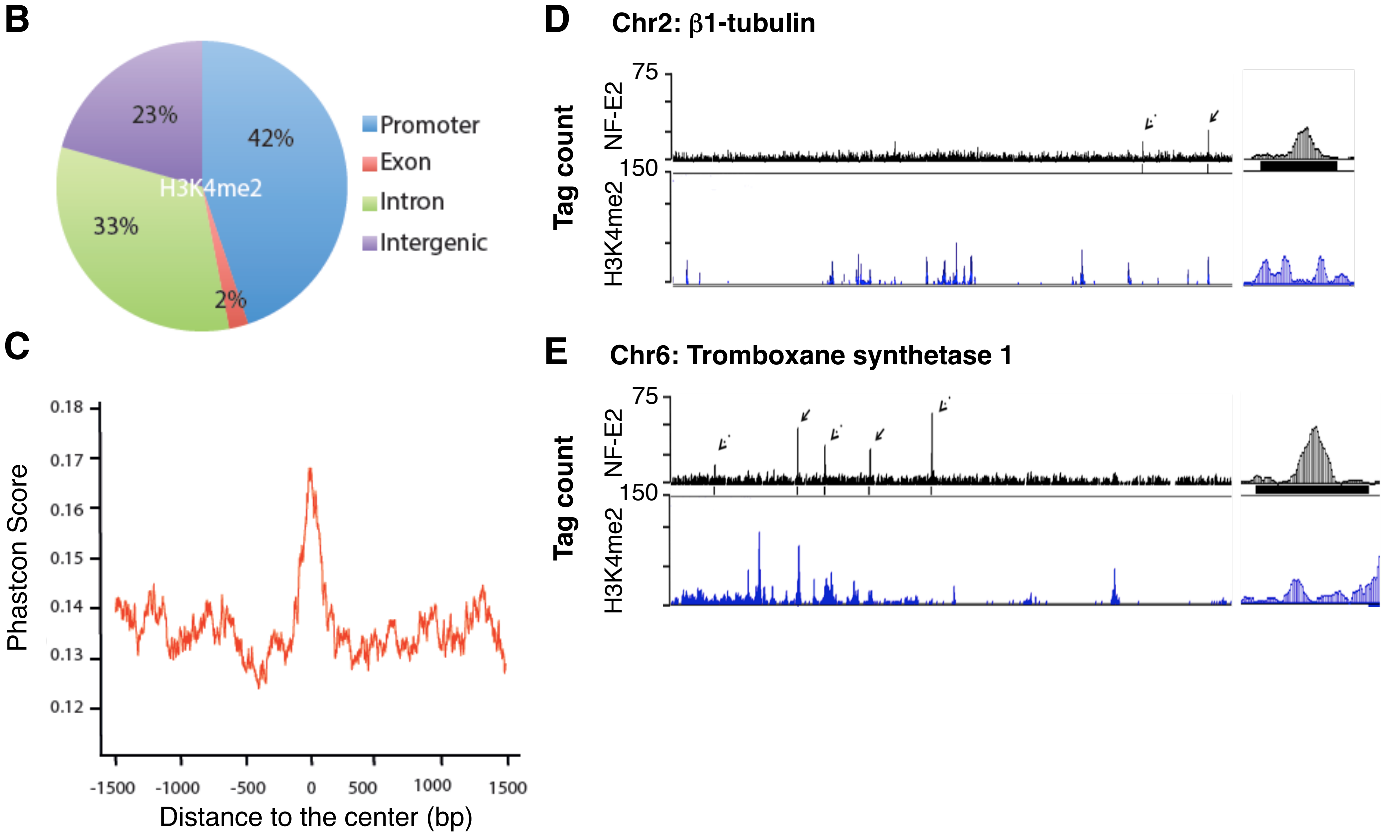

Suppl. Figure 2

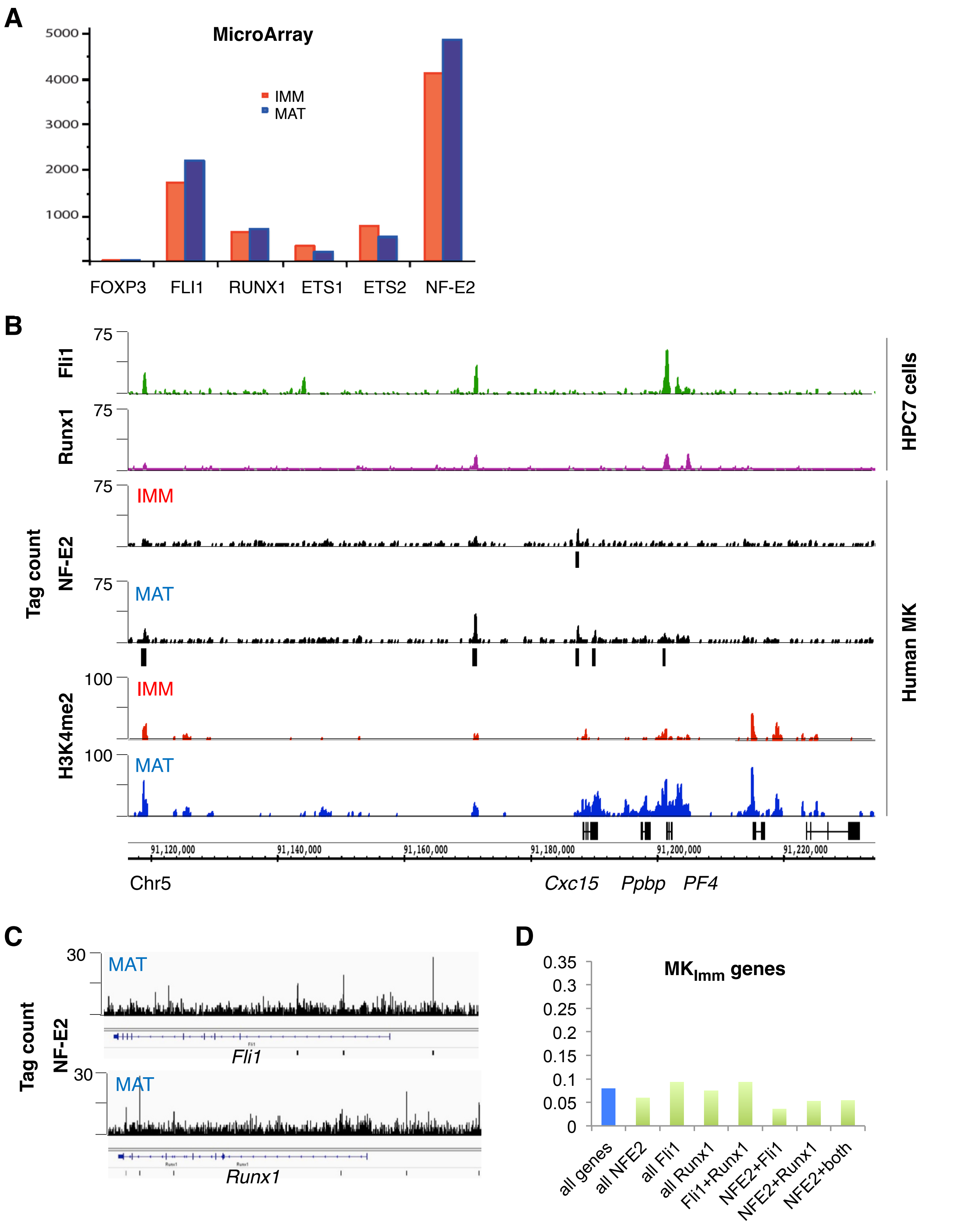

Supplementary Table 1. Differential expression and TF regulation pattern of 692 genes that are up-regulated during MK maturation

RefSeq ID	Gene Symbol	MK_MAT log2FC	MK_MAT p-value	NFE2 depen-dency	NFE2 count <20kb	nearest NFE2 (bp)	from IMM	co-FLI1	co-RUNX1	nearest FLI1 (bp)	nearest RUNX1 (bp)
NM_010045	Darc	5.090	1.730E-05	-1	0	21736	1	0	0	138	4215
NM_008293	Hsd3b1	5.239	6.520E-05	-1	1	2669	1	0	0	281512	4482
NM_008221	Hbb-y	6.531	1.708E-04	1	1	7887	1	1	1	6659	7917
NM_001114087	Pdlim7	2.503	4.256E-04	-1	1	2942	1	1	0	145	234
NM_001164557	Pdzk1ip1	3.691	4.256E-04	1	1	14973	0	1	1	7107	2141
NM_010484	Slc6a4	6.164	5.022E-04	-1	1	7544	0	1	0	2056	5539
NM_010016	Cd55	2.222	5.168E-04	-1	1	17130	0	0	0	337	45807
NM_013750	Phlda3	2.570	7.995E-04	0	0	128415	0	1	0	405	42964
NM_024479	Wbscr27	1.820	7.995E-04	0	1	18367	0	0	0	62	19175
NM_172118	Myl9	5.480	7.995E-04	0	1	8666	0	0	0	34300	22100
NM_007657	Cd9	1.906	8.292E-04	0	2	11178	0	0	0	4799	8334
NM_133187	Fam198b	2.513	8.997E-04	0	0	80920	0	1	0	76	43
NM_011150	Lgals3bp	2.656	9.627E-04	0	0	401171	1	0	0	2	10338
NM_001160404	Galnt1	1.547	9.627E-04	0	1	4040	0	0	0	485	44884
NM_025331	Gng11	3.603	9.627E-04	-1	0	86642	1	0	0	111	111
NM_013885	Clic4	1.454	9.627E-04	0	0	32957	0	0	0	170	30
NM_028064	Slc39a4	3.238	9.627E-04	-1	1	527	0	1	0	569	4277
NM_013492	Clu	3.668	9.627E-04	0	1	2813	0	0	0	2187	2224
NM_029001	Elovl7	1.853	9.627E-04	-1	0	28713	0	0	0	27481	5602
NM_001177751	Tsc22d1	2.690	1.064E-03	-1	1	9604	0	0	0	17650	6066
NM_009025	Rasa3	2.235	1.215E-03	-1	0	63180	0	1	0	1102	60028
NM_018762	Gp9	3.832	1.215E-03	-1	0	60784	0	1	0	108	58
NM_001161724	Ilk	1.338	1.215E-03	-1	0	1305424	0	0	0	102	74321
NM_009101	Rras	1.490	1.215E-03	0	1	15352	0	0	0	598	539
NM_001001892	H2-K1	1.375	1.510E-03	0	1	5164	0	0	0	49	27450
NM_146006	Lss	1.166	1.524E-03	0	0	44897	0	1	0	89	103614
NM_007440	Alox12	4.228	1.524E-03	-1	1	4998	0	1	0	532	1238
NM_001077361	Fhl1	3.425	1.524E-03	-1	0	188140	0	0	0	9648	2709

NM_207648	H2-gs10	2.239	1.524E-03	0	0	122965	0	0	0	92178	85368
NM_001159633	Slc44a1	2.016	1.546E-03	0	0	38414	0	0	0	1343	8483
NM_022029	Nrgn	3.444	1.697E-03	-1	0	22256	0	1	0	829	4105
NM_001113527	Isg20	3.182	1.697E-03	-1	0	290991	0	0	0	291	15792
NM_025508	Gmpr	1.442	1.764E-03	0	0	51714	0	1	0	21	12936
NM_011708	Vwf	4.146	1.811E-03	-1	1	12602	0	0	0	51	133
NM_138583	D16H22S680E	1.089	1.827E-03	0	1	551	0	0	0	11	1492
NM_134133	2010002N04Rik	2.688	1.827E-03	-1	1	540	1	1	1	514	418
NM_008615	LOC677317	1.751	1.875E-03	0	0	109125	1	0	1	72016	47412
NM_026617	Tmbim4	1.451	1.949E-03	-1	1	128	0	1	1	89	132
NM_009303	Syngr1	1.321	1.949E-03	0	1	7810	1	1	0	7688	9052
NM_008185	Gstt1	4.322	1.949E-03	0	0	244284	1	0	0	33701	2787
NM_001032378	Pecam1	1.595	2.077E-03	-1	1	1047	0	0	0	11	4461
NM_026178	LOC100047565	1.782	2.077E-03	-1	2	6408	0	1	0	356	23417
NM_053149	Hemgn	2.053	2.138E-03	0	0	159846	1	1	0	29	11956
NM_018738	Igtp	2.660	2.138E-03	-1	2	5200	0	0	0	6088	13300
NM_176848	Fbxo2	4.745	2.199E-03	-1	1	4500	0	0	0	543	31875
NM_021430	Rilpl1	1.566	2.199E-03	0	0	55979	1	1	1	1499	9029
NM_001168256	Tmem40	2.184	2.267E-03	-1	2	2895	0	0	0	470	3247
NM_001025364	Rtn2	3.344	2.267E-03	0	0	23809	0	1	0	4853	19194
NM_019985	Clec1b	4.831	2.289E-03	-1	2	8969	0	1	0	16	10948
NM_009450	Tubb2a	1.323	2.289E-03	0	1	14	0	1	0	7	11482
NM_008207	H2-T24	2.945	2.289E-03	-1	1	18475	0	1	0	142	41330
NM_175414	Tspan9	2.714	2.289E-03	-1	0	40862	0	1	0	6364	173739
NM_001145801	Ctla2b	3.047	2.351E-03	-1	1	138	0	1	0	78	39271
NM_001048207	Gypc	1.598	2.381E-03	0	0	105584	0	0	0	9	7982
NM_019932	Pf4	1.294	2.420E-03	0	3	327	0	0	0	8	22
NM_026527	Chac2	1.797	2.462E-03	-1	0	170850	0	1	0	139	15832
NM_134076	Abhd4	1.357	2.462E-03	0	3	668	0	0	0	5181	5195
NM_001001999	Gp1bb	1.331	2.469E-03	0	0	32500	0	0	0	675	2749
NM_144520	Sec14l2	2.663	2.746E-03	0	0	114172	0	1	0	1041	968
NM_001109991	Col18a1	3.024	2.791E-03	0	0	93042	0	1	0	17036	33221

NM_001163574	Tjp1	2.434	2.908E-03	0	0	343279	0	0	0	153	2318
NM_026849	Mtmr14	1.079	2.908E-03	-1	0	89648	0	0	0	62	12743
NM_027763	Treml1	3.640	2.952E-03	-1	0	60555	0	0	0	748	694
NM_001163704	Fbxo6	1.301	2.954E-03	0	1	4241	0	0	0	119	40617
NM_025661	Ormdl3	2.179	2.975E-03	0	0	84821	0	0	0	1	324
NM_029934	Mboat7	1.130	2.975E-03	0	0	443382	0	1	0	31	1176
NM_133687	Cxxc5	1.164	2.975E-03	0	0	64226	1	0	0	1244	1157
NM_172753	Csgalnact1	1.423	2.975E-03	-1	0	173741	0	0	0	13307	5287
NM_018804	Syt11	2.403	2.975E-03	0	0	54592	1	0	0	14504	44174
NM_026418	Rgs10	2.114	3.000E-03	0	1	14843	0	1	0	3475	27810
NM_001145959	Ndrg2	2.260	3.043E-03	-1	1	1753	0	1	0	26	6379
NM_010326	Gp1ba	3.714	3.043E-03	-1	1	22	0	1	1	71	176
NM_001130412	Lpin1	1.160	3.043E-03	0	0	263772	0	0	0	1813	6882
NM_019699	Fads2	1.532	3.043E-03	0	0	72142	1	0	0	337	175
NM_010170	F2rl2	2.795	3.043E-03	-1	2	180	0	0	1	1143	111
NM_001040654	Cdkn2a	2.913	3.043E-03	0	0	46040	0	0	0	3458	9627
NM_029219	Rnf19b	1.988	3.043E-03	-1	1	9929	0	0	0	9031	78
NM_053195	Slc24a3	2.718	3.332E-03	-1	0	45897	0	0	0	8533	37217
NM_173006	Pon3	1.629	3.357E-03	0	0	186588	0	0	0	24	26437
NR_015608	1810058I24Rik	1.459	3.429E-03	0	0	132629	0	0	0	29	332
NM_008681	Ndrg1	1.873	3.429E-03	-1	1	12499	0	0	0	422	24604
NM_008967	Ptgir	2.896	3.429E-03	-1	2	673	0	0	0	2044	9426
NM_145148	Frmd4b	2.017	3.429E-03	-1	0	207532	0	1	0	86426	3984
NM_001170911	Prr13	1.330	3.433E-03	-1	1	1981	0	1	0	63	997
NM_011734	Siae	0.873	3.433E-03	0	0	83357	0	1	0	86	124
NR_003513	Neat1	2.038	3.433E-03	-1	6	58	0	0	1	1221	215
NM_172507	Sh3bgrl2	3.154	3.433E-03	-1	2	1831	0	1	0	1794	19981
NM_007976	F5	2.791	3.433E-03	-1	2	13861	0	0	0	2632	1738
NM_172647	F11r	1.538	3.433E-03	0	0	56659	0	0	0	4047	2896
NM_001081050	Pard3b	1.817	3.433E-03	-1	0	108465	0	0	0	8758	3100
NM_010394	H2-Q7	2.496	3.433E-03	0	0	137243	0	0	0	77900	99646
NM_011385	Ski	1.003	3.598E-03	0	0	82141	0	0	0	948	929

NM_197986	Tmem140	3.601	3.600E-03	0	1	6481	1	1	0	28	11564
NM_023275	Rhoj	2.379	3.600E-03	-1	1	16554	0	0	0	11	4502
NM_018810	Mkrn1	2.253	3.600E-03	-1	3	4342	0	0	0	65	5203
NM_053090	Fam126a	1.492	3.600E-03	-1	0	23248	0	0	0	24	57
NM_026432	Tmem66	0.862	3.600E-03	0	2	22	1	1	0	27	2174
NM_011401	Slc2a3	2.505	3.600E-03	-1	1	16171	0	1	1	42	52
NM_008397	Itga6	1.925	3.600E-03	0	1	4088	0	0	0	156	8776
NM_011539	Tbxas1	2.614	3.600E-03	-1	2	43	1	1	0	81	5770
NM_016780	Itgb3	3.005	3.600E-03	-1	1	3771	0	1	1	95	373
NM_010941	Nsdhl	1.121	3.600E-03	0	0	422700	0	0	0	523	29860
NM_178869	Ttll1	1.065	3.600E-03	0	1	15583	0	0	0	53	75431
NM_029688	Srxn1	1.747	3.600E-03	-1	1	284	1	1	0	210	11850
NM_001083922	Wbp1	0.762	3.600E-03	0	0	93723	0	0	0	378	5932
NM_001111049	Cd151	1.383	3.600E-03	0	1	1654	0	1	0	1686	73495
NM_008871	Serpine1	2.535	3.600E-03	1	0	30884	1	1	1	4049	3467
NM_029465	Clec4g	1.330	3.600E-03	-1	0	307587	0	0	0	8783	4421
NM_023279	Tubb3	4.725	3.600E-03	-1	1	1335	0	0	0	37194	5336
NM_007642	Cd28	1.760	3.600E-03	-1	0	114384	0	0	0	43608	25496
NM_027127	Gpx8	1.298	3.600E-03	0	0	371979	0	1	0	55782	11229
NM_001048054	Dusp16	1.235	3.610E-03	0	0	38977	0	0	0	12	1635
NM_008148	Gp5	3.200	3.610E-03	-1	1	19	0	1	0	70	5076
NM_023738	Uba7	1.916	3.621E-03	0	1	12562	0	0	0	84	7330
NM_001159301	Lgals9	1.606	3.621E-03	0	0	66561	0	1	1	35	3820
NM_001081490	Fbxo9	0.816	3.630E-03	0	1	14	0	1	0	41	7238
NM_009894	Cideb	1.529	3.630E-03	0	0	34867	0	0	0	688	19931
NM_007791	Csrp1	0.866	3.630E-03	0	0	91527	0	1	0	78	6076
NM_133167	LOC676024	1.835	3.631E-03	-1	1	4397	0	0	0	1184	3815
NM_024233	Rexo2	0.822	3.631E-03	0	0	123387	0	1	0	14772	7038
NM_019983	Rabgef1	1.225	3.681E-03	0	1	15573	0	1	1	15490	1730
NM_001113419	Schip1	1.144	3.681E-03	0	0	246244	0	1	0	246239	8159
NM_007609	Casp4	1.373	3.742E-03	-1	1	9657	0	0	0	113	62175
NM_001033167	Slc22a23	3.042	3.742E-03	-1	3	3555	1	1	0	3489	801

NM_009375	Tg	3.408	3.742E-03	-1	0	102821	0	1	0	14107	30523
NM_001161420	Vldlr	2.815	3.742E-03	0	0	89832	0	0	0	67295	29621
NM_023785	Ppbp	3.775	3.875E-03	0	3	3589	0	0	0	2119	3894
NM_001136484	Gcnt1	1.436	3.913E-03	-1	1	4316	0	1	1	180	4250
NM_001145799	Ctla2a	2.471	3.913E-03	-1	2	212	0	1	1	98	152
NM_139269	Pla2g16	1.711	3.945E-03	0	1	74	0	0	0	23823	4742
NM_134250	Havcr2	2.872	4.177E-03	-1	1	141	0	1	0	128	7126
NM_001136062	Eno3	1.086	4.277E-03	0	1	18068	0	1	1	3624	2714
NM_001110275	Itsn1	1.592	4.286E-03	-1	0	60451	0	1	1	616	89
NM_001130188	Sgce	1.809	4.294E-03	-1	0	31490	0	0	0	135142	28705
NM_001111099	Cdkn1a	1.372	4.296E-03	0	2	4587	0	1	0	4596	11713
NM_033612	Cela1	1.926	4.308E-03	-1	2	13803	0	1	1	744	13911
NM_001166377	Armcx1	2.617	4.333E-03	0	0	65598	0	1	0	192	25792
NM_008702	Nlk	1.291	4.333E-03	-1	0	96703	0	0	0	91	13637
NM_008771	P2rx1	1.781	4.333E-03	0	0	39846	0	0	0	225	172
NM_009250	Serpini1	2.314	4.333E-03	-1	0	135710	0	0	0	648	372
NM_144521	Snap47	0.889	4.333E-03	0	0	31070	0	1	0	28334	28309
NM_011563	Prdx2	0.782	4.417E-03	0	0	55535	1	0	0	2801	6978
NM_001111073	Fxyd5	0.695	4.424E-03	0	0	622167	0	1	0	352	15202
NM_001172099	Cuedc1	1.738	4.424E-03	-1	1	7255	0	1	1	5026	5076
NM_145925	Pttg1ip	1.502	4.460E-03	-1	2	2483	0	0	0	2067	2112
NM_001037098	Nacc2	1.814	4.460E-03	0	0	24030	1	0	1	5301	531
NM_146094	Fads1	0.909	4.460E-03	0	1	9241	1	0	0	5549	244
NM_001025613	Otud7b	2.498	4.523E-03	-1	0	40108	0	0	0	9	32
NM_001113460	Tec	1.689	4.570E-03	-1	3	4854	0	1	0	316	506
NM_009908	Cmas	1.641	4.583E-03	0	1	616	1	0	0	1024	47855
NM_007553	Bmp2	2.248	4.587E-03	-1	0	39443	0	0	0	55228	1541
NM_019819	Dusp14	3.440	4.632E-03	-1	0	164856	0	0	0	5359	61204
NM_011449	Spa17	1.400	4.654E-03	-1	0	83125	0	1	0	145	107
NM_019418	Tnfsf14	2.667	4.704E-03	-1	2	784	0	0	0	66	22861
NM_133911	Gpr125	0.955	4.704E-03	0	0	1656891	0	0	0	111	18620
NM_019549	Plek	2.170	4.719E-03	0	1	3622	0	1	1	83	3491

NM_011596	Atp6v0a2	0.991	4.719E-03	0	0	154226	1	1	1	65	27
NM_009105	Rsu1	1.342	4.719E-03	0	1	6299	0	0	0	92	2730
NM_025572	2610528J11Rik	2.906	4.719E-03	-1	0	25740	0	0	0	10309	11506
NM_013792	Naglu	1.112	4.722E-03	0	2	6202	0	1	0	143	44788
NM_019547	Rbm38	1.048	4.795E-03	0	1	16330	1	1	0	21	23
NM_028127	Frmd6	0.977	4.795E-03	0	0	149466	0	1	0	62	3045
NR_033498	AI504432	1.965	4.795E-03	-1	0	546588	0	1	0	1212	244
NM_030700	LOC100046560	1.220	4.836E-03	0	0	856569	0	0	0	685	16975
NM_001163310	Rit1	1.040	4.836E-03	-1	1	1153	1	0	0	286	11358
NM_008640	Laptm4a	0.708	4.836E-03	0	0	399665	0	1	0	574	69171
NM_001033189	C77080	0.884	4.836E-03	1	1	15162	1	1	0	8196	8103
NM_011527	Tal1	1.707	4.871E-03	0	1	2143	0	0	0	478	1489
NM_016867	Gipc2	1.751	4.968E-03	0	0	38371	0	0	0	5540	2871
NM_011817	Gadd45g	2.468	5.012E-03	0	1	1577	0	0	0	38	132
NM_133697	1110003E01Rik	1.595	5.012E-03	0	0	46157	0	0	0	199	59
NM_001135657	Ptprj	1.213	5.012E-03	-1	0	76937	0	0	0	9918	18907
NM_008135	Slc6a9	0.743	5.054E-03	0	0	117641	0	0	0	345	1030
NM_001038015	Gnpda2	0.791	5.054E-03	0	1	8274	0	0	0	35393	136019
NM_018882	Gpr56	1.673	5.056E-03	0	3	73	0	0	0	945	14202
NM_008287	Hrsp12	1.673	5.056E-03	0	2	2300	0	1	0	2367	325
NM_018869	Grk5	1.346	5.120E-03	-1	0	50464	0	1	0	506	56460
NM_001142916	Plod2	0.889	5.136E-03	0	0	54867	0	0	0	167128	10254
NM_019440	Irgm2	2.384	5.158E-03	-1	1	3498	0	0	0	124	9449
NM_001038700	Fnbp1	0.715	5.235E-03	0	3	6043	0	0	0	68	3009
NM_021454	Cdc42ep5	1.474	5.303E-03	-1	0	27791	0	1	0	59	56903
NM_001109045	Aqp8	1.763	5.303E-03	0	0	124975	0	0	0	63193	77085
NM_011597	Tjp2	0.971	5.311E-03	0	0	58492	0	1	0	106	39520
NM_080555	Ppap2b	2.225	5.311E-03	0	0	50542	0	0	0	5599	25688
NM_026272	Narf	1.246	5.473E-03	0	0	91185	0	0	0	6	9024
NM_001164248	Tpm1	0.884	5.473E-03	0	1	11291	0	0	0	5405	5434
NM_138651	Cds2	1.225	5.473E-03	0	1	6148	0	1	0	6079	10417
NM_025797	Cyb5	1.091	5.625E-03	0	2	2294	1	1	0	2319	26503

NM_001172154	Dnase1l1	1.212	5.839E-03	-1	0	143053	1	0	0	357	23264
NM_011804	Creg1	1.442	5.839E-03	0	2	80	0	0	0	419	2640
NM_011815	Fyb	2.005	5.904E-03	-1	1	10472	0	1	0	5114	5092
NM_009808	Casp12	2.912	5.907E-03	-1	1	2337	0	1	0	2099	25559
NM_010184	Fcer1a	3.050	5.926E-03	-1	0	64031	0	0	0	102136	25033
NM_013730	Slamf1	2.601	6.057E-03	-1	1	5130	0	1	0	51	114
NM_030024	Prr15	1.379	6.057E-03	0	0	166147	0	1	0	853	26522
NM_001033210	Pls1	3.521	6.057E-03	-1	1	17992	0	1	0	12281	307
NM_011633	Traf5	1.561	6.057E-03	0	0	80001	0	0	0	13916	21083
NM_012048	Polk	0.903	6.088E-03	0	0	138973	0	0	0	53	70
NM_173742	Rnasek	1.195	6.088E-03	0	1	10490	0	1	0	236	2014
NM_153781	Pygb	0.821	6.088E-03	-1	1	13985	0	1	0	63	14317
NM_016916	Blcap	1.103	6.088E-03	0	1	1038	0	0	0	385	24867
NM_001047435	Pard6a	0.686	6.088E-03	0	0	50261	0	1	0	546	371
NM_010282	Ggps1	0.647	6.088E-03	0	1	12139	0	0	0	733	7209
NM_133655	Cd81	1.210	6.088E-03	0	1	9551	0	1	0	3281	13026
NM_021381	Prokr1	1.750	6.088E-03	-1	1	7189	0	1	0	6605	2526
NM_010689	Lat	1.914	6.110E-03	0	0	52410	0	1	0	18	1996
NM_010575	Itga2b	2.707	6.122E-03	0	0	24335	0	1	0	300	359
NM_013610	Ninj1	0.982	6.132E-03	0	2	2608	0	1	0	564	518
NM_011170	Prnp	2.294	6.243E-03	0	0	25972	0	0	0	96	21448
NM_016754	Mylpf	1.015	6.285E-03	-1	1	7413	0	0	0	384	2908
NM_025944	2810432L12Rik	1.717	6.285E-03	0	0	339788	0	0	0	326	733
NM_001122739	Inppl1	0.799	6.285E-03	0	0	23389	0	0	0	476	454
NM_001038695	Kdm3a	0.703	6.285E-03	0	0	104633	0	1	0	498	79
NR_003368	Pvt1	0.656	6.285E-03	0	0	26582	0	0	0	8459	12338
NM_145602	Ndrg4	2.727	6.285E-03	-1	0	149444	0	1	0	12839	8294
NM_030595	Nbea	2.429	6.285E-03	0	0	56194	1	0	0	116842	7329
NM_007901	S1pr1	0.893	6.290E-03	0	0	25415	0	1	1	36	20090
NM_013690	Tek	1.944	6.367E-03	0	0	136059	0	1	0	29595	42937
NM_009906	Tpp1	0.897	6.382E-03	0	0	1321042	0	0	0	669	58703
NM_001171052	Mta3	0.926	6.382E-03	0	0	26116	1	1	1	649	53

NM_001111060	Cd59a	2.305	6.382E-03	0	0	50347	1	1	0	2829	64
NM_001109761	Capn3	2.294	6.382E-03	-1	0	76043	0	0	0	7525	46627
NM_008969	Ptgs1	1.317	6.640E-03	0	0	29105	0	1	0	661	19940
NM_009551	Zfand5	1.114	6.651E-03	-1	2	702	0	1	0	431	6243
NM_001159883	Dnajb2	1.896	6.651E-03	-1	1	17680	1	0	0	7104	19552
NM_007635	Ccng2	0.734	6.713E-03	-1	0	38118	0	0	0	642	27419
NM_010165	Eya2	2.224	6.831E-03	-1	0	22026	0	0	0	109	5115
NM_027230	Zmynd8	1.048	6.845E-03	0	1	669	1	1	0	936	4071
NM_021897	Trp53inp1	0.736	6.927E-03	0	0	67637	1	0	0	285	4
NM_053267	Selm	0.753	6.961E-03	0	0	718200	0	1	0	20876	6480
NM_001166642	Bcas3	0.937	6.969E-03	0	0	26144	0	0	0	4	142
NM_133983	Cd276	1.306	6.969E-03	0	1	1106	0	1	0	1039	1607
NM_020590	Gabarapl1	1.251	6.969E-03	0	2	488	0	0	0	1090	63278
NM_001001491	Tpm4	1.849	6.969E-03	0	1	9281	0	0	0	1557	1769
NM_023456	Npy	4.860	6.969E-03	-1	0	34269	0	0	0	16000	107398
NM_021347	Gsdma	1.013	6.969E-03	0	1	7716	0	0	0	18168	18227
NM_145575	Cald1	2.377	7.194E-03	0	0	27644	0	0	0	34890	3912
NM_009506	Vegfc	1.666	7.196E-03	0	0	44608	0	1	0	12	12396
NM_008772	P2ry1	2.565	7.233E-03	0	0	69802	0	0	0	12	54
NM_026004	Nt5c3	1.602	7.233E-03	-1	2	4375	0	1	0	75	153
NM_009344	Phlda1	1.704	7.316E-03	0	1	1532	0	0	0	184	1068
NM_008928	Map2k3	0.605	7.453E-03	0	1	3499	1	1	0	81	62
NM_001037741	Gpx4	0.821	7.453E-03	0	3	1796	0	0	0	483	55
NM_011767	Zfr	0.600	7.453E-03	0	0	109601	0	1	0	233	10692
NM_145486	LOC100044703	1.980	7.500E-03	0	0	88979	0	0	0	126	6526
NM_153789	Mylip	1.297	7.500E-03	0	0	66012	0	1	0	128	1395
NM_008535	Lyl1	0.599	7.500E-03	0	0	23937	0	0	0	1408	220
NM_021537	Stk25	1.067	7.532E-03	-1	1	14045	0	0	0	1110	6531
NM_019579	Mpp5	0.941	7.553E-03	0	2	553	0	1	0	643	7621
NM_172570	Trim47	1.542	7.627E-03	0	1	5469	0	1	0	762	11818
NM_016676	Rab10	0.631	7.627E-03	0	0	24346	1	0	0	230	5057
NM_021878	Jarid2	1.248	7.632E-03	0	0	281307	0	0	0	202	1017

NM_016672	Ddc	2.722	7.790E-03	0	1	1178	0	1	0	1169	17630
NM_025480	Tmem128	0.711	7.866E-03	0	0	239906	1	0	0	26	81
NM_177876	Vps37b	0.898	7.904E-03	0	1	4698	0	1	0	31	47
NM_010149	Epor	1.435	7.904E-03	0	0	39359	0	1	1	904	928
NM_001128606	Epb4.1	1.209	7.984E-03	0	1	18831	1	1	0	5705	327
NM_010815	Grap2	2.060	8.010E-03	0	2	15803	0	1	0	164	12836
NM_009599	Ache	1.956	8.010E-03	0	0	96500	1	1	0	731	6189
NM_026441	Pef1	0.712	8.010E-03	0	1	4934	1	0	0	39	343
NM_024495	Car13	1.066	8.010E-03	0	0	424159	0	0	0	135	1861
NM_001002267	Tmem158	1.908	8.010E-03	-1	1	14818	0	1	0	10945	1050
NM_001077696	Hdac5	0.711	8.024E-03	0	0	54947	0	0	0	81	40
NM_021880	Prkar1a	0.728	8.096E-03	0	0	177127	0	1	0	23	15045
NM_172691	B230312A22Rik	1.383	8.096E-03	-1	0	59595	0	0	0	1167	431
NM_001005608	Itgb4	0.780	8.096E-03	0	0	49189	0	1	1	29896	8278
NM_011653	Gm3756	0.862	8.113E-03	0	0	76228	0	0	0	143	35092
NM_021428	Dexi	0.596	8.113E-03	0	0	133879	0	1	0	2170	562
NM_010228	Flt1	1.560	8.113E-03	0	0	135197	0	0	0	4394	4252
NM_008398	Itga7	0.823	8.113E-03	0	1	2597	0	0	0	10754	6788
NM_029083	Ddit4	1.779	8.113E-03	-1	0	49529	0	0	0	15839	51538
NM_001177778	Dlg3	0.917	8.113E-03	0	0	470635	0	0	0	46457	9063
NM_026148	Lims1	1.364	8.154E-03	0	0	23237	0	0	0	41	93
NM_133234	Bbc3	1.602	8.154E-03	-1	0	31266	0	1	0	942	1268
NM_054045	Hist1h3a	4.257	8.280E-03	-1	0	30444	0	1	0	1494	18328
NR_030716	5430417L22Rik	1.852	8.280E-03	-1	0	86479	1	1	1	152	248
NM_015806	Mapk6	1.174	8.280E-03	0	0	46111	0	0	0	244	185
NM_138656	Mvd	0.944	8.280E-03	0	0	27658	0	0	0	202	18018
NM_009624	Adcy9	2.588	8.280E-03	-1	0	46403	0	0	0	5100	805
NM_001136065	Hipk2	0.939	8.309E-03	0	1	6114	0	0	0	1936	8726
NM_033144	8-Sep	1.121	8.329E-03	0	0	137287	0	0	1	952	53227
NM_001122892	Fyn	0.665	8.383E-03	0	0	95377	0	0	0	976	86
NM_011580	Thbs1	1.889	8.571E-03	0	0	45211	0	1	0	65	862
NM_010311	Gnaz	2.237	8.582E-03	-1	0	136972	0	0	0	2892	2121

NM_175133	1110038D17Rik	0.737	8.803E-03	0	1	14383	0	0	0	590	12403
NM_153795	Fermt3	0.966	8.803E-03	0	1	946	0	0	0	1279	16
NM_018854	Ift20	0.594	8.870E-03	-1	0	64286	0	0	0	107	24995
NM_013586	Loxl3	0.651	8.902E-03	0	1	19556	1	0	0	832	56390
NM_008089	Gata1	1.807	8.902E-03	0	0	32929	0	0	0	3515	7463
NM_008719	Npas2	1.477	9.144E-03	-1	0	100808	0	0	0	1502	13133
NM_026793	Myct1	2.114	9.150E-03	-1	0	22375	0	1	0	59	55
NM_138677	Edem1	0.993	9.150E-03	-1	1	14143	0	0	0	106	984
NM_001081212	Irs2	2.348	9.150E-03	-1	0	40527	0	0	0	1137	583
NM_138602	Praf2	1.026	9.188E-03	0	0	37424	1	1	0	6630	8001
NM_031877	Wasf1	0.957	9.236E-03	0	0	35337	0	0	0	284	15
NM_133858	Fam63a	1.480	9.279E-03	-1	1	10849	1	1	1	355	11021
NM_001177833	Smox	1.690	9.279E-03	0	0	48407	1	0	0	4681	62
NM_008685	Nfe2	0.921	9.285E-03	-1	1	16779	0	0	0	21	2531
NM_013589	Ltbp2	2.402	9.285E-03	0	0	459215	1	1	0	21948	52179
NM_009899	Clca1	3.361	9.285E-03	0	0	72301	0	0	0	40268	54573
NM_001033208	Gcom1	1.449	9.286E-03	0	0	204752	0	0	0	14468	65112
NM_011595	Timp3	1.877	9.365E-03	0	0	246966	0	1	0	181	14532
NM_024207	Derl1	1.183	9.365E-03	-1	1	14428	1	1	0	54	849
NM_001038999	Atp8a1	1.239	9.365E-03	-1	0	31473	0	0	0	216	335
NM_001109992	Ptpn11	0.946	9.365E-03	0	1	6048	0	0	0	81	11945
NM_027102	Esam	2.297	9.365E-03	0	2	2400	0	1	0	2271	4126
NM_001111110	Cmah	1.374	9.365E-03	-1	0	58896	0	0	0	11001	21698
NM_011347	Selp	2.105	9.365E-03	-1	2	11963	0	1	1	11845	11878
NM_001025610	Ms4a7	3.095	9.451E-03	1	0	265993	0	0	0	14	55894
NM_028841	Tspan17	1.389	9.618E-03	0	0	324667	0	0	0	174	23921
NM_001172424	Dhrs3	1.842	9.687E-03	0	0	23889	0	0	0	2776	2822
NM_027324	Sfxn1	0.589	9.687E-03	0	0	29458	0	1	0	9108	998
NM_001039546	Myo6	1.084	9.694E-03	-1	0	1569060	0	0	0	43	526
NM_011099	Pkm2	0.589	9.694E-03	0	2	2625	0	0	0	265	189
NM_001012306	Hsd3b2	1.702	9.694E-03	0	0	94714	1	0	0	345817	84721
NM_008783	Pbx1	1.005	9.779E-03	0	1	14905	0	0	0	186	56441

NM_008706	Nqo1	2.023	9.910E-03	0	2	431	1	1	0	369	16320
NM_001159536	Adcy3	0.679	9.910E-03	0	1	5797	0	0	0	50818	13956
NM_007975	F2rl3	2.081	1.013E-02	0	0	78473	0	1	0	363	3124
NM_007899	Ecm1	2.174	1.015E-02	0	1	12615	0	0	0	18	23879
NM_145619	Parp3	0.990	1.023E-02	0	0	231116	0	0	0	442	9251
NM_001163336	Atp2a3	1.271	1.023E-02	-1	2	1870	0	0	0	685	7092
NM_001160145	Tmem9	1.231	1.023E-02	0	0	338656	0	0	0	6716	9899
NM_008859	Prkcq	2.147	1.027E-02	0	0	24746	0	0	0	119	16338
NM_153057	Nomo1	1.166	1.027E-02	0	1	2018	0	1	0	71	23741
NM_001025245	Mbp	1.212	1.039E-02	-1	1	2763	0	1	0	2757	3382
NM_001077184	Bsg	0.667	1.046E-02	0	1	19768	1	0	0	22498	13479
NM_016903	Esd	0.899	1.049E-02	0	1	1212	0	1	0	74	4096
NM_139300	Mylk	1.954	1.049E-02	0	1	47	0	1	0	123	3169
NM_001082553	Rab27b	2.823	1.054E-02	-1	0	40025	0	1	0	448	38135
NM_011101	Prkca	2.189	1.058E-02	0	1	7592	0	0	0	332	18595
NM_138741	Sdpr	2.960	1.058E-02	0	0	23877	0	0	0	7234	11319
NM_009112	S100a10	0.863	1.067E-02	0	0	762425	0	0	0	1087	26351
NM_028133	Egln3	1.277	1.067E-02	-1	1	15858	0	0	1	12466	43
NM_001111279	Wdfy1	0.963	1.071E-02	0	0	264215	0	1	1	548	153
NM_173755	Ube2o	1.920	1.071E-02	-1	4	621	0	0	0	1317	62
NM_029555	Gstk1	0.646	1.073E-02	0	0	53599	0	0	0	18790	6726
NM_145100	Lypd1	2.206	1.073E-02	0	0	127888	0	0	0	248949	5524
NM_020626	Tmem27	2.636	1.075E-02	0	1	2557	0	0	0	2619	5627
NM_028011	Tom1l1	1.717	1.079E-02	0	1	11714	0	1	0	29	49406
NM_001160406	Gfi1b	1.263	1.080E-02	0	0	23722	1	0	1	8	560
NM_011421	Smpd1	0.952	1.080E-02	0	0	1123194	0	0	0	14	5
NM_026056	Cap2	2.247	1.080E-02	0	1	9072	0	0	0	95	5455
NM_145384	Pqlc2	1.032	1.080E-02	0	0	76102	0	0	0	413	2972
NM_024211	Slc25a11	0.776	1.080E-02	0	1	7849	0	1	1	513	7149
NM_028135	LOC100047091	2.351	1.088E-02	0	1	17789	0	0	0	650	2559
NM_023732	Abcb6	0.940	1.088E-02	0	1	864	1	1	0	871	7731
NM_001113423	Slain2	1.403	1.093E-02	0	0	31714	0	1	0	20	3174

NM_146168	Vopp1	1.116	1.097E-02	0	0	21833	0	0	0	649	11901
NM_139144	Ogt	0.756	1.111E-02	0	0	260179	0	0	0	78	144
NM_001164268	Kalrn	1.318	1.125E-02	0	0	42046	0	0	0	171627	22001
NM_030113	Arhgap10	0.737	1.129E-02	0	0	75226	0	0	1	169	44519
NM_021409	Pard6b	0.713	1.129E-02	0	0	21983	0	0	0	385	11038
NM_001039090	Skil	1.213	1.132E-02	0	0	33685	0	1	1	440	494
NM_001163753	Rab37	0.915	1.133E-02	0	0	125855	0	1	0	19251	16881
NM_019464	Sh3glb1	0.671	1.136E-02	0	0	31885	0	0	0	147	14157
NM_028814	2810403A07Rik	0.629	1.138E-02	0	0	20887	0	0	0	46	26
NM_023324	Peli1	0.904	1.138E-02	0	1	8474	0	1	0	149	522
NM_025486	Tmem208	0.705	1.138E-02	0	1	13708	0	0	0	48	18714
NM_026693	Gabarapl2	0.695	1.143E-02	0	2	797	0	0	0	73	21980
NM_029870	A930001N09Rik	0.716	1.144E-02	0	0	174514	0	0	0	29	446
NM_001128080	Tspan32	1.162	1.161E-02	0	0	38152	0	1	0	1505	42
NM_027460	Slc25a33	0.888	1.164E-02	0	1	6603	1	1	0	6514	36335
NM_019718	Arl3	0.718	1.170E-02	0	0	117271	0	0	0	106	111
NM_028030	Rbpms2	1.729	1.174E-02	-1	1	1200	0	1	0	1225	20569
NM_011123	Plp1	1.903	1.174E-02	0	0	1932997	0	0	0	81089	81136
NM_010786	Mdm2	0.838	1.180E-02	0	1	7082	0	1	0	2	152
NM_011452	Serpinb9b	1.561	1.199E-02	1	1	790	0	0	0	20951	126920
NM_009127	Scd1	2.532	1.199E-02	-1	0	95501	0	0	0	630	51301
NM_009109	Ryr1	0.747	1.222E-02	0	0	247796	0	0	0	9650	2214
NM_001038602	Marveld2	1.424	1.226E-02	0	0	112604	0	0	0	17452	12558
NM_181820	Tmc4	0.980	1.239E-02	0	0	459354	0	1	0	2178	6474
NM_001122683	Bdh1	0.635	1.241E-02	0	0	65074	0	0	0	322	3672
NM_008786	Pcmt1	0.588	1.241E-02	0	0	121731	0	0	0	520	641
NM_001159401	Upp1	1.037	1.244E-02	0	0	93040	0	0	0	91	8
NM_023716	Tubb2b	2.058	1.253E-02	0	0	52331	0	1	0	28300	19229
NM_001001326	St5	1.235	1.273E-02	0	0	38005	0	0	0	11894	53721
NM_001166408	Rai14	0.786	1.273E-02	0	1	9060	0	1	0	61	37071
NM_001083315	St7	1.141	1.277E-02	-1	3	262	0	1	1	170	286
NM_011777	Zyx	1.257	1.302E-02	0	3	906	0	0	0	52	102

NM_008808	Pdgfa	0.868	1.308E-02	0	0	89536	0	0	0	29744	2698
NM_011896	LOC100046643	2.118	1.315E-02	-1	0	32618	0	1	0	90	3464
NM_026058	Lass4	0.654	1.346E-02	0	0	254714	1	0	1	8	36
NM_010826	Mrvi1	3.543	1.351E-02	-1	2	4386	0	0	0	1622	10492
NM_016965	Nckap1	1.992	1.351E-02	0	1	16172	0	0	0	693	781
NM_013520	Flt3l	1.044	1.357E-02	-1	0	26153	0	0	0	1191	87
NM_025635	Zwint	0.922	1.360E-02	0	2	437	0	0	0	134	18796
NM_144865	Reep2	1.252	1.367E-02	0	0	113814	0	1	0	1046	13986
NM_024432	Ubxn6	0.597	1.373E-02	0	0	43917	0	1	0	69	37
NM_181039	Lphn1	0.648	1.373E-02	0	0	159958	0	0	1	96	126
NM_032398	Plvap	1.105	1.373E-02	0	0	63481	0	0	0	49	21914
NM_019980	Litaf	0.762	1.393E-02	0	0	27844	0	0	0	865	2396
NM_181411	Aftph	1.314	1.395E-02	0	0	155078	0	0	0	89	14
NM_001146100	Hk1	0.759	1.399E-02	0	0	109700	0	0	0	991	129
NM_133838	Ehd4	0.795	1.404E-02	0	0	162947	0	0	0	943	9565
NM_007722	Cxcr7	2.260	1.404E-02	-1	0	71170	1	0	0	263449	10071
NM_013835	Trove2	1.719	1.417E-02	-1	0	43489	0	0	0	71	2026
NM_001042743	Mast2	0.666	1.417E-02	0	0	61281	1	0	0	172	57495
NM_019976	Psrc1	0.761	1.417E-02	0	0	29099	0	0	0	5485	38646
NM_010060	Dnahc11	0.659	1.417E-02	0	1	9297	1	0	0	22320	68241
NM_009026	Rasd1	1.684	1.445E-02	-1	0	78961	0	1	1	31	45393
NM_001029841	Sla	1.557	1.445E-02	0	0	150311	0	0	0	6277	62849
NM_029983	Sla2	1.783	1.447E-02	0	1	13270	0	0	0	187	216
NM_032465	Cd96	1.805	1.455E-02	-1	0	475604	0	1	0	22	10668
NM_008409	Itm2a	1.450	1.455E-02	0	0	1213802	0	1	0	152728	50011
NM_001130174	Tnnt2	1.354	1.467E-02	1	0	198716	0	1	0	8266	53018
NM_011987	Pla2g10	2.455	1.487E-02	-1	0	36032	0	1	0	518	15932
NM_013757	Sytl4	0.917	1.487E-02	-1	0	318686	0	0	0	77525	6268
NM_001042487	Dlgap4	0.909	1.510E-02	0	0	25199	0	0	0	1121	794
NM_025926	Dnajb4	1.892	1.511E-02	-1	0	45150	1	0	0	21	379
NM_052976	Ophn1	1.926	1.511E-02	-1	1	13414	0	1	1	59	13523
NM_145130	Lpcat3	0.644	1.511E-02	0	0	29693	0	1	0	29	16992

NM_001162532	Fam174b	1.368	1.511E-02	-1	0	180100	0	0	0	25020	20355
NM_001045489	Mfge8	0.794	1.515E-02	1	0	56165	0	0	0	19	25371
NM_007920	Elf1	0.688	1.522E-02	-1	0	55055	0	0	0	134	116
NM_026677	Rab13	1.475	1.523E-02	0	0	58964	0	0	0	7163	16373
NM_028238	Rab38	1.239	1.581E-02	0	1	12014	0	0	0	18	26583
NM_001146180	Mtss1	1.201	1.590E-02	0	0	25369	0	1	0	1354	16893
NM_201357	Tssc1	0.779	1.609E-02	0	2	1016	0	0	0	59	115915
NM_030725	Syt13	2.386	1.609E-02	-1	0	33761	0	0	0	3991	68181
NM_020010	Cyp51	0.976	1.649E-02	0	0	231003	0	0	0	115	30
NM_146126	Sord	1.150	1.653E-02	-1	2	807	1	0	0	8111	7990
NM_018761	Ctnnal1	1.511	1.657E-02	0	0	72099	0	0	0	152	3437
NM_001110273	Slc14a2	1.044	1.657E-02	-1	1	16796	0	0	0	6707	36101
NM_026740	Slc46a1	0.741	1.657E-02	0	1	18342	0	0	0	43855	32110
NM_019482	Panx1	0.656	1.662E-02	-1	2	7621	0	1	0	2	27148
NM_025649	Mad2l1bp	1.039	1.665E-02	0	1	14768	0	1	0	17	69
NM_027406	Aldh1l1	1.109	1.674E-02	0	0	21225	0	0	0	54927	150585
NM_013489	Cd84	1.668	1.680E-02	0	2	5042	0	0	0	33	6483
NM_001077638	Prmt2	0.990	1.680E-02	0	0	69015	0	1	0	115	44264
NM_001168471	Dynll2	0.728	1.682E-02	0	1	5984	0	0	0	49308	9443
NM_009502	Vcl	1.609	1.705E-02	0	0	83934	0	0	0	318	290
NM_025760	Ptplad2	1.581	1.705E-02	-1	1	955	1	1	0	950	31859
NM_008150	Gpc4	2.380	1.710E-02	0	0	1169479	0	0	0	225437	47466
NM_009270	Sqle	0.863	1.711E-02	0	0	43441	0	1	0	214	55880
NM_133888	Smpdl3b	1.043	1.750E-02	0	0	21587	0	0	0	3235	47219
NM_025785	Fbxo25	0.675	1.764E-02	0	0	127099	0	0	0	61	40
NM_010262	Gbx2	2.098	1.767E-02	-1	0	31768	0	1	1	1919	7728
NM_023305	Ubap1	0.712	1.775E-02	0	3	3935	0	0	0	88	12682
NM_181821	Hcfc1r1	0.626	1.780E-02	0	0	129205	0	1	1	48	75299
NM_008129	Gclm	1.703	1.794E-02	0	2	68	1	1	1	32	57
NM_026738	1110007C09Rik	0.982	1.794E-02	0	1	15231	1	1	0	2209	988
NM_008679	Ncoa3	1.014	1.794E-02	0	1	9605	0	1	0	8888	3541
NM_008880	Plscr2	2.339	1.803E-02	0	0	129423	0	0	0	965	1351

NM_001002011	Lmna	1.187	1.806E-02	0	3	7997	0	1	0	43	6876
NM_009657	Aldoc	1.294	1.806E-02	-1	0	123160	0	0	0	4166	21767
NM_008385	Inpp5b	0.856	1.812E-02	0	0	45908	0	1	0	7974	30889
NM_009325	Tbxa2r	1.129	1.815E-02	0	0	29826	0	0	0	1704	737
NM_001042451	Snca	2.407	1.826E-02	0	0	22884	0	0	0	827	564
NM_022410	Myh9	1.229	1.840E-02	-1	2	7626	0	1	0	4285	127
NM_021896	Gucy1a3	1.849	1.840E-02	0	0	83948	0	1	0	4864	12
NM_001177841	Otub2	0.611	1.840E-02	0	0	102839	0	1	0	5021	37235
NM_008576	Abcc1	0.829	1.844E-02	0	3	4671	0	0	0	191	32519
NM_145562	Parm1	1.140	1.844E-02	0	0	23291	0	0	0	215324	39997
NM_008571	Mcpt2	3.899	1.852E-02	-1	0	163943	0	0	0	51443	257133
NM_001163552	Ap4b1	0.764	1.852E-02	0	1	9842	1	1	0	28	92
NM_001110147	Tnk2	0.766	1.852E-02	0	0	44369	0	1	0	6548	286
NM_029649	Tmem101	0.755	1.860E-02	0	0	128712	0	0	0	16	52590
NM_019521	Gas6	1.249	1.860E-02	1	0	100524	0	1	0	94671	1602
NM_017379	Tuba8	1.495	1.860E-02	0	2	4250	0	1	1	206	4219
NM_028375	Cxx1c	1.586	1.862E-02	0	0	2562477	0	0	0	187372	83110
NM_009743	Bcl2l1	0.802	1.865E-02	0	0	42695	0	1	1	0	860
NM_009761	Bnip3l	0.679	1.865E-02	0	0	89454	0	0	0	148	25
NM_011738	Ywhah	0.693	1.865E-02	0	0	254570	0	0	0	2005	2783
NM_027144	Arhgef12	1.296	1.868E-02	0	1	3125	1	0	0	479	423
NM_145521	Ppapdc3	1.230	1.868E-02	0	0	34339	0	1	0	5722	7100
NM_172601	Rab2b	0.654	1.868E-02	0	0	175205	0	1	0	138	21493
NM_009177	St3gal1	1.129	1.868E-02	0	2	3999	0	0	0	45	23548
NM_133859	Olfml3	0.765	1.883E-02	1	2	7970	0	0	0	407	3502
NM_009931	Col4a1	0.659	1.883E-02	0	0	54129	0	0	0	10109	45551
NM_008740	Nsf	0.806	1.905E-02	0	0	47253	0	1	0	10054	4854
NM_028176	Cda	0.602	1.905E-02	1	1	11339	0	0	0	22904	63206
NM_013605	Muc1	1.931	1.907E-02	0	0	192558	1	0	0	767	3619
NM_010761	Ccndbp1	0.728	1.907E-02	0	0	74391	0	0	0	2	4881
NM_011104	Prkce	0.809	1.941E-02	-1	0	20234	0	1	0	200	20869
NM_025768	Grtp1	1.248	1.941E-02	0	1	8255	1	0	0	13038	295

NM_016852	Wbp2	0.910	1.960E-02	0	2	93	0	1	0	7	1743
NM_001159616	Pigp	0.796	1.965E-02	0	0	108668	0	0	0	7	19650
NM_175684	Fchsd1	0.783	1.965E-02	0	0	86030	1	1	0	22	18553
NM_018797	LOC100044106	1.091	1.965E-02	0	1	4844	0	0	0	3818	1666
NM_013738	Plek2	0.726	1.965E-02	0	0	118460	0	0	0	4028	4154
NM_001159593	Slc20a1	0.965	1.990E-02	0	1	8162	0	0	0	243	48066
NM_001029936	Cytsb	1.566	1.991E-02	0	0	54718	0	0	0	298	611
NM_178785	Rasal3	0.804	2.001E-02	0	1	4955	0	1	1	652	615
NM_009242	Sparc	1.216	2.008E-02	1	0	48911	0	0	0	2147	15764
NM_138753	Hexim1	1.016	2.042E-02	0	0	130323	0	0	0	159	16551
NM_007737	Col5a2	0.638	2.042E-02	0	0	357248	1	1	0	292130	67672
NM_013723	Podxl	1.259	2.048E-02	0	0	52154	0	0	1	28941	17088
NM_001136084	Tph1	0.896	2.072E-02	0	0	79794	0	0	0	14874	5526
NM_028454	Tm7sf2	1.213	2.080E-02	-1	0	34475	0	0	0	6697	17029
NM_027356	Ufsp1	1.867	2.139E-02	0	0	102892	1	1	0	163	202
NM_008795	Cdk18	0.725	2.139E-02	1	0	125075	0	0	0	3594	6031
NM_001177776	Ahi1	0.836	2.141E-02	0	2	11496	0	0	0	13876	4780
NM_013784	Pign	0.966	2.143E-02	0	0	231222	0	0	0	466	333
NM_023422	Gm11277	2.497	2.153E-02	-1	0	74836	1	0	0	632	54675
NM_007670	Cdkn2b	1.898	2.153E-02	0	0	60659	1	1	0	25386	2457
NM_026252	Cpeb4	1.416	2.178E-02	0	0	47377	0	0	0	1416	774
NM_008548	Man1a	1.193	2.179E-02	-1	2	95	0	1	0	34	41559
NM_019447	Hgfac	1.617	2.206E-02	1	1	5605	0	1	1	5606	5558
NM_021422	Dnaja4	0.939	2.218E-02	0	0	57817	0	0	0	16602	35032
NM_008608	Mmp14	0.874	2.235E-02	0	0	47204	0	1	0	2081	4844
NM_007743	Col1a2	1.439	2.247E-02	0	1	11689	0	0	0	48683	6236
NM_181569	Nprl3	0.653	2.277E-02	0	2	2313	0	1	1	12	2347
NM_001145779	Kif2a	0.839	2.283E-02	0	3	2392	1	0	0	3646	280
NM_011785	Akt3	1.267	2.283E-02	0	0	364094	0	0	0	9847	51647
NM_001170537	Mef2c	0.945	2.284E-02	0	0	56360	0	0	0	14347	11479
NM_013838	Trpc6	2.900	2.289E-02	-1	0	155905	0	0	0	3	49966
NM_024229	Pcyt2	0.751	2.289E-02	0	3	5093	0	0	0	296	7119

NM_009695	Apoc2	0.616	2.289E-02	1	0	124026	0	0	0	9207	12487
NM_011500	Strn	0.712	2.291E-02	0	1	11680	0	1	0	227	226
NM_019919	Ltbp1	2.501	2.298E-02	-1	0	53257	0	0	0	552	665
NM_172471	Itih5	0.953	2.313E-02	0	0	41732	0	0	0	759	25792
NM_172779	Ddx26b	0.866	2.316E-02	-1	0	465258	0	0	0	84	176100
NM_001146153	Homer3	1.071	2.342E-02	0	1	9927	1	1	0	184	30574
NM_019760	Serinc1	0.781	2.342E-02	0	0	449937	0	0	0	22	8763
NM_178618	Fam83g	0.609	2.342E-02	0	1	2959	0	0	0	31178	52834
NM_009029	Rb1	1.549	2.379E-02	0	0	24695	0	0	0	514	11132
NM_025436	Sc4mol	0.826	2.397E-02	0	0	326762	0	0	0	281	7608
NM_026921	Isca1	0.977	2.421E-02	0	1	81	0	1	1	156	187
NM_177150	Cenpt	0.684	2.453E-02	0	1	10159	0	1	0	292	1898
NM_133764	Atp6v0e2	0.928	2.479E-02	1	0	453542	0	1	0	15374	16674
NM_027238	Ttc39b	1.477	2.494E-02	0	0	49757	0	0	0	3933	39415
NM_025745	Erlec1	0.601	2.507E-02	0	0	180893	0	0	0	247	48066
NM_027571	P2ry12	2.095	2.534E-02	-1	0	34870	0	1	0	1869	8291
NM_175184	Fam125b	0.865	2.552E-02	0	1	19394	0	0	0	3010	18482
NM_053198	Sfxn4	0.608	2.554E-02	-1	0	42527	1	0	0	14	42187
NM_008235	Hes1	1.683	2.559E-02	0	1	2678	1	0	0	1014	1719
NM_028207	Dusp3	0.946	2.560E-02	0	0	31528	0	0	0	655	46
NM_019472	Myo10	0.698	2.569E-02	0	1	17946	0	0	0	296	37450
NM_011309	S100a1	0.807	2.599E-02	0	0	46243	0	1	0	69	14069
NM_001164661	Cyfip1	0.772	2.607E-02	0	1	11244	0	0	0	115	2478
NM_001039515	Arl4a	0.730	2.613E-02	-1	1	10428	1	0	0	1187	35387
NM_011173	Pros1	1.173	2.626E-02	0	0	2341633	0	0	0	110	7230
NM_173752	1110067D22Rik	2.670	2.632E-02	-1	0	65598	0	1	1	38016	503
NM_010432	Hipk1	0.832	2.642E-02	-1	1	8398	1	1	0	84	1263
NM_018729	Cd244	1.572	2.648E-02	-1	0	64972	0	0	0	2171	2144
NM_013463	Gla	1.218	2.714E-02	-1	0	51333	0	1	0	182	18521
NM_001001181	BC031181	0.652	2.714E-02	0	1	16528	0	0	0	156	108141
NM_026428	Dcxr	0.803	2.716E-02	0	0	53669	1	0	0	13603	54137
NM_013482	Btk	0.963	2.720E-02	-1	0	69198	0	1	0	111	656

NM_010398	C920025E04Rik	1.827	2.720E-02	0	0	30616	0	1	0	8932	53471
NM_010769	Matn1	0.941	2.725E-02	1	0	30235	0	1	1	23152	25909
NM_024208	Echdc3	0.718	2.772E-02	0	1	324	0	1	0	336	7288
NM_007527	Bax	0.683	2.776E-02	0	3	5922	1	1	1	371	5828
NM_001033634	Zyg11b	0.646	2.780E-02	0	1	17753	0	1	0	660	65193
NM_001009818	11-Sep	1.083	2.781E-02	0	0	32407	0	0	0	218	3021
NM_030743	Rnf114	0.630	2.781E-02	0	0	148590	0	0	0	217	2914
NM_001039373	Mtcp1	0.766	2.781E-02	0	0	257575	0	1	0	13696	61463
NM_001083120	Enah	0.666	2.796E-02	0	0	29920	0	0	0	51326	32293
NM_001161796	Gucy1b3	2.712	2.819E-02	0	1	12782	0	1	0	13	15810
NM_013876	Rnf11	1.207	2.827E-02	0	1	4691	1	0	0	2373	34
NM_011851	Nt5e	1.233	2.851E-02	0	0	35095	0	0	0	2771	40851
NM_011643	Trpc1	1.968	2.851E-02	-1	1	4713	0	0	0	51739	51562
NM_009679	Ap2m1	0.603	2.888E-02	0	1	12714	1	0	0	231	4669
NM_144822	Cbara1	0.819	2.900E-02	0	1	5165	1	0	0	114	2831
NM_029409	Mff	0.709	2.902E-02	0	0	67492	0	0	0	74	2987
NM_001162904	Mdm1	1.197	2.905E-02	0	0	91261	0	1	0	32040	31190
NM_010169	F2r	1.358	2.913E-02	0	1	227	0	1	1	141	81
NM_008624	Mras	0.623	2.913E-02	0	0	179695	1	0	0	120994	10642
NM_025943	Dzip1	0.696	2.933E-02	0	0	120816	0	0	0	14511	235
NM_007837	Ddit3	2.089	2.933E-02	-1	0	179623	0	0	0	219	234
NM_001159965	Ralgps2	0.991	2.933E-02	0	0	72000	0	0	0	444	719
NM_008845	Pip4k2a	0.620	2.933E-02	0	0	24712	0	0	0	227	20527
NM_001077411	Gba	0.612	2.933E-02	0	0	218682	1	0	0	59	14010
NM_197989	Fam58b	0.642	2.941E-02	0	0	151007	0	0	0	20372	0
NM_007823	Cyp4b1	0.989	2.966E-02	0	0	147653	0	0	0	84705	11460
NM_001040684	Hsd3b7	0.609	3.009E-02	0	1	19589	0	0	0	29	17660
NM_007711	Clcn3	0.819	3.013E-02	0	1	61	1	1	0	204	25760
NM_138646	Hps4	1.038	3.028E-02	0	0	100378	0	1	0	45	9161
NM_020332	Ank	1.046	3.058E-02	0	0	150530	0	0	0	2616	94
NM_024477	Ttc28	0.822	3.059E-02	-1	0	28844	0	0	0	39802	57125
NM_144819	Ccdc92	0.679	3.065E-02	0	0	210317	0	0	0	493	34817

NM_029530	6330527O06Rik	0.632	3.065E-02	0	0	39230	0	1	0	39351	22978
NM_145076	Trim24	0.681	3.078E-02	0	1	7805	0	0	0	19	160
NM_016671	Il27ra	0.840	3.085E-02	0	0	148401	0	0	0	23915	23816
NM_001164117	Serpinb6a	1.478	3.120E-02	0	0	141939	0	1	0	28424	14882
NM_138587	Fam3c	0.710	3.170E-02	0	0	228113	0	0	0	104	81
NM_053272	Dhcr24	0.715	3.177E-02	-1	0	52747	1	1	0	31115	48409
NM_025980	Nrarp	0.698	3.181E-02	0	1	7856	0	0	0	16030	16044
NM_011018	Sqstm1	0.742	3.181E-02	0	2	302	0	0	0	496	11077
NM_001130525	LOC100048460	0.708	3.183E-02	0	0	40238	0	0	0	687	323
NM_026209	1810063B07Rik	0.625	3.183E-02	0	0	395517	0	1	0	458	3038
NM_009038	Rcvrn	0.756	3.184E-02	0	0	159886	0	0	0	87024	41797
NM_001081175	Itpkb	0.802	3.195E-02	0	1	17390	0	0	0	83	91
NM_026735	Mobkl1a	1.396	3.195E-02	0	1	3409	0	1	0	31	9698
NM_008330	Ifi47	0.942	3.216E-02	0	0	37308	1	0	0	718	9979
NM_009373	Tgm2	0.978	3.216E-02	1	0	52915	0	0	0	4226	8563
NM_198294	Tanc1	1.083	3.216E-02	0	0	189031	0	1	0	30848	2751
NM_133986	Tcta	0.668	3.257E-02	0	0	71125	0	0	0	155	1207
NM_008590	Mest	3.696	3.268E-02	-1	1	1285	0	0	0	25409	1502
NM_007465	Birc2	0.608	3.268E-02	0	0	70481	0	0	0	1678	1732
NM_010739	Muc13	1.075	3.317E-02	0	0	61828	0	0	0	474	32389
NM_028013	Endod1	1.091	3.419E-02	0	0	178431	0	0	0	149	12613
NM_011676	Unc119	0.921	3.419E-02	0	0	103836	0	0	0	735	2443
NM_009895	Cish	0.799	3.419E-02	0	1	16228	0	0	0	27	15707
NM_021344	LOC100047138	3.083	3.419E-02	-1	0	332411	0	1	0	20734	27327
NM_010612	Kdr	1.356	3.424E-02	0	0	512459	0	1	1	129	534
NM_008376	Gimap1	0.904	3.428E-02	-1	0	454631	0	0	0	54	8661
NM_145508	Dyrk3	1.027	3.431E-02	-1	2	845	0	0	0	116	3222
NM_145425	AV249152	0.682	3.431E-02	-1	0	170876	0	0	0	389	21727
NM_001025395	Src	1.812	3.431E-02	0	0	27564	0	0	0	4593	6504
NM_145360	Idi1	1.067	3.466E-02	0	0	129074	1	0	0	14540	14475
NM_007602	Capn5	0.640	3.491E-02	0	0	207097	0	0	0	6941	9368
NM_013748	Clnk	1.584	3.491E-02	0	0	38545	0	0	0	11891	21665

NM_011920	Abcg2	1.131	3.493E-02	0	0	244694	1	0	0	274	25076
NM_007780	Csf2rb	0.596	3.493E-02	0	1	7432	0	0	0	11	54390
NM_009517	Zmat3	1.923	3.499E-02	1	0	52228	0	0	0	52	8742
NM_153175	Gimap6	0.812	3.510E-02	-1	0	485433	0	0	0	21	4177
NM_010117	Rhbdf1	1.012	3.510E-02	0	0	23529	1	0	0	2663	5362
NM_009707	Arhgap6	1.715	3.519E-02	-1	1	9840	0	0	0	15610	27415
NM_009696	Apoe	1.479	3.556E-02	1	0	101724	0	0	0	3843	4264
NM_020490	Ltb4r2	0.919	3.556E-02	0	0	38366	0	0	0	4187	23065
NM_001142952	Fam46c	3.532	3.570E-02	-1	0	48965	0	0	1	787	14466
NM_001083934	Myom1	1.783	3.590E-02	0	0	21544	0	0	1	148	16347
NM_023348	Snap29	0.620	3.599E-02	-1	0	156024	0	0	0	44	64
NM_026346	Fbxo32	1.423	3.602E-02	0	0	69108	0	1	0	17939	524
NM_007689	Chad	1.960	3.648E-02	0	0	32383	0	0	0	485	31921
NM_010554	Il1a	2.580	3.667E-02	0	0	46605	0	0	0	4153	7335
NM_011203	Ptpn12	0.904	3.670E-02	0	1	2070	0	0	0	443	695
NM_023160	Cml1	0.660	3.670E-02	0	0	303867	0	0	0	5	45761
NM_139272	Galnt2	0.907	3.681E-02	-1	1	6726	0	0	0	3145	25205
NM_009686	Apbb2	0.733	3.688E-02	0	0	295353	0	1	0	94403	11677
NM_025865	2310030G06Rik	2.095	3.793E-02	1	0	198223	0	0	1	21656	21637
NM_020050	Tmem9b	0.803	3.798E-02	0	0	44664	0	0	0	6	179
NM_138598	D11Wsu99e	0.761	3.798E-02	0	1	2987	1	0	0	89	191
NM_007781	Csf2rb	1.195	3.805E-02	1	1	12953	0	0	0	15389	34004
NM_146057	Dap	0.666	3.838E-02	0	2	111	1	1	1	123	49
NM_015783	Gm9706	2.140	3.838E-02	0	1	2788	0	1	0	1838	10170
NM_001145831	Kifc3	1.137	3.838E-02	0	0	129867	0	1	0	38	37115
NM_009665	Amd1	0.689	3.838E-02	-1	1	9346	0	0	1	33	9351
NM_001113569	Stxbp1	0.836	3.838E-02	0	0	70488	0	0	0	10422	89907
NM_175093	Trib3	1.261	3.861E-02	-1	1	5888	0	1	0	89	718
NM_009983	Ctsd	1.159	3.873E-02	0	2	22	1	1	0	154	7292
NM_022019	Dusp10	1.350	3.902E-02	0	0	40463	0	0	0	49	1543
NM_009141	Cxcl5	2.007	3.946E-02	0	4	940	1	0	0	13	1583
NM_008987	Ptx3	0.900	3.946E-02	0	0	20772	0	1	0	20829	22184

NM_033541	Oas1c	0.939	3.967E-02	0	0	86561	1	0	0	16	36691
NM_008605	Mmp12	3.599	3.980E-02	1	0	168748	0	0	0	125792	10450
NM_001038609	Mapt	0.629	3.983E-02	0	0	46958	0	0	0	801	36048
NM_021461	Mknk1	0.908	3.993E-02	0	1	16197	0	1	1	113	1524
NM_025432	Trappc2	1.332	4.000E-02	0	0	342722	0	0	0	3873	150960
NM_178204	Hist1h3a	0.943	4.000E-02	-1	2	3300	0	0	0	4440	5642
NM_019790	Tmeff2	0.673	4.000E-02	0	1	7730	0	1	0	7740	12940
NM_028331	C1qtnf6	0.838	4.000E-02	0	1	18907	1	0	0	13571	32984
NM_001159577	Lnx1	0.683	4.000E-02	0	0	231881	0	0	0	167196	25177
NM_053254	Tle6	0.605	4.003E-02	0	1	9239	0	0	0	112	26689
NM_145216	Rasl10a	1.170	4.039E-02	0	1	7419	0	1	0	3798	3653
NM_001159424	Il12a	1.827	4.079E-02	0	2	13426	0	1	1	1597	13542
NM_013549	Hist2h2aa1	2.537	4.079E-02	-1	0	29278	0	1	0	2660	18737
NM_007598	Cap1	0.732	4.081E-02	0	2	1668	0	0	0	15	1
NM_080448	Srgap3	0.990	4.081E-02	-1	0	105635	0	0	0	8699	8710
NM_001029895	Ate1	0.686	4.117E-02	0	1	9491	0	0	0	352	24742
NM_001168525	Sgms1	0.714	4.126E-02	0	0	44294	0	1	0	6657	30378
NM_009794	Capn2	1.536	4.160E-02	0	2	324	0	0	0	4374	13090
NM_001112796	Bicd1	0.657	4.163E-02	0	0	215144	1	1	0	4523	9695
NM_010119	Ehd1	0.820	4.165E-02	0	0	20203	1	0	0	805	965
NM_008147	Gp49a	1.384	4.169E-02	0	0	47578	0	0	0	124	9925
NM_175353	Exoc6	0.665	4.169E-02	0	1	11293	0	1	1	9452	11339
NM_145541	Rap1a	0.681	4.221E-02	0	1	10029	0	0	0	141	11827
NM_010020	Slc6a3	1.467	4.225E-02	0	0	271924	0	0	0	36319	42064
NM_029519	Rap2a	0.860	4.233E-02	0	0	40658	0	0	0	699	406
NM_145441	Gm6245	0.660	4.237E-02	0	0	517981	0	0	0	9724	9794
NM_026126	Fundc2	0.593	4.313E-02	0	0	291720	0	1	0	71	95608
NM_010273	Gdi1	0.919	4.325E-02	0	0	120375	1	0	0	7859	586
NM_008570	Mcpt1	2.762	4.328E-02	-1	0	139784	0	0	0	71523	232974
NM_001013833	Prkg1	1.941	4.332E-02	-1	0	145611	0	1	0	541	659
NM_025378	Ifitm3	0.587	4.403E-02	0	0	48089	0	0	0	2338	11146
NM_010789	Meis1	1.212	4.406E-02	0	0	48921	0	1	0	9	47848

NM_198011	9130019O22Rik	0.729	4.419E-02	0	0	120402	0	0	0	109	41885
NM_021883	Tmod1	0.772	4.428E-02	1	0	158825	0	0	0	67096	116
NM_025638	Gdpd1	0.987	4.457E-02	0	0	30881	0	0	0	10	46613
NM_177231	Arrb1	0.796	4.488E-02	0	2	2116	0	0	0	463	51894
NM_009878	Cdkn2d	0.839	4.516E-02	0	0	54597	0	0	1	3938	41382
NM_145484	Zfp758	0.660	4.554E-02	0	0	905758	0	0	0	62730	96725
NM_025858	Shisa5	0.708	4.611E-02	0	1	11382	0	0	0	74	93
NM_177632	Fam43a	1.391	4.611E-02	-1	0	133917	0	0	0	56	3376
NM_176837	Arhgap18	1.315	4.615E-02	-1	0	27240	0	1	1	96	11761
NM_009419	Tpst2	0.675	4.664E-02	0	0	33990	0	1	0	233	97
NM_029494	Rab30	1.091	4.684E-02	0	0	183430	0	0	0	187	578
NM_025960	Trappc6a	0.586	4.707E-02	0	1	8370	0	0	0	416	43598
NM_013659	Sema4b	0.672	4.719E-02	-1	0	20511	0	0	0	11535	4845
NM_019811	Acss2	0.922	4.721E-02	-1	0	50019	0	0	0	116	16
NM_008331	Ifit1	2.254	4.745E-02	0	1	10277	0	0	0	7230	12764
NM_001079908	Fgfr1	0.771	4.745E-02	1	0	53108	0	0	0	73507	83
NM_001110850	Crem	0.876	4.769E-02	0	0	66877	0	1	0	157	1960
NM_008120	Gja4	0.909	4.769E-02	0	0	330096	0	0	0	58	114271
NM_029478	Tmem49	0.596	4.811E-02	0	0	25778	1	0	0	12	15450
NM_007405	Adcy6	1.364	4.852E-02	0	0	75173	0	0	0	1379	31333
NM_018784	St3gal6	1.702	4.920E-02	0	1	4717	0	0	0	2682	89395
NM_144813	Slc24a1	1.566	4.978E-02	0	0	98312	0	0	0	9232	9200
NM_010879	LOC100044475	1.385	4.991E-02	-1	0	31869	0	0	0	493	14198
NM_009579	Slc30a1	1.090	4.991E-02	-1	0	26366	1	1	0	26421	23303
